# Forecasting stock prices with a feature fusion LSTM-CNN model using different representations of the same data

**DOI:** 10.1371/journal.pone.0212320

**Published:** 2019-02-15

**Authors:** Taewook Kim, Ha Young Kim

**Affiliations:** 1 Department of Financial Engineering, Ajou University, Yeongtong-gu, Suwon, Republic of Korea; 2 Department of Data Science, Ajou University, Yeongtong-gu, Suwon, Republic of Korea; Universidad Veracruzana, MEXICO

## Abstract

Forecasting stock prices plays an important role in setting a trading strategy or determining the appropriate timing for buying or selling a stock. We propose a model, called the feature fusion long short-term memory-convolutional neural network (LSTM-CNN) model, that combines features learned from different representations of the same data, namely, stock time series and stock chart images, to predict stock prices. The proposed model is composed of LSTM and a CNN, which are utilized for extracting temporal features and image features. We measure the performance of the proposed model relative to those of single models (CNN and LSTM) using SPDR S&P 500 ETF data. Our feature fusion LSTM-CNN model outperforms the single models in predicting stock prices. In addition, we discover that a candlestick chart is the most appropriate stock chart image to use to forecast stock prices. Thus, this study shows that prediction error can be efficiently reduced by using a combination of temporal and image features from the same data rather than using these features separately.

## Introduction

Forecasting stock prices is an attractive pursuit for investors and researchers who want to beat the stock market. However, forecasting stock prices is difficult. Cowles [1] showed that no skills existed to predict the stock market. Fama [2] proposed efficient market theory, which states that a stock price already reflects all new information related to the stock and implies that no one can beat the market because stock prices are already set fairly. Contrary to this theory, many attempts have been made to predict stock prices to obtain profits using various techniques [3, 4, 5, 6] since determining the market timing for buying or selling a stock at a certain price is an important part of a trading strategy [7].

Taking an econometric approach, Keim et al. [6] showed that predicting stock returns with statistical significance was partially possible using a model with several predetermined variables. French et al. [8] proposed using the generalized autoregressive conditional heteroscedasticity (GARCH) model to predict stock prices using the relationship between a stock’s volatility and its return. Fama and French [9] proposed two-factor models using size and book-to-market equity, both of which are related to a company’s fundamental information, to predict stock prices. Jeantheau [10] suggested that under stationary conditions, the autoregressive conditional heteroskedastic model could be applied to predict stock prices. Ariyo et al. [11] proposed a predictive model for stock prices using the autoregressive integrated moving average model. They found that this model had the potential to predict short-term future stock prices on the New York Stock Exchange and the Nigeria Stock Exchange.

When using an econometric method, it is advantageous to have explanatory power because this power derives the results given the theoretical background. However, the assumptions used in econometric models do not necessarily hold in the real world. Artificial neural networks (ANNs), by contrast, are not limited by these assumptions and can detect nonlinear relationships in the characteristics of data. Thus, many scholars have been studying the prediction of stock prices based on these models. Kimoto et al. [12] proposed a prediction system for forecasting stocks listed on the Tokyo Stock Exchange using a system of ANNs and achieved excellent profits using the prediction system in a simulation exercise. Kim et al. [13] used a genetic algorithms approach to optimize thresholds for feature discretization and connection weights between layers to forecast a stock price index. The results indicated that this approach outperformed conventional neural networks. Tsang et al. [4] proposed a buying and selling alert system using ANNs to forecast Hong Kong stock prices. This system showed that the overall hit ratio was over 70%. Yudong et al. [14] suggested a forecasting model using improved bacterial chemotaxis optimization and a back propagation ANN. They showed that the proposed model was superior to traditional back propagation ANNs for predicting the S&P 500 index. Wang et al. [15] proposed a wavelet denoising-based back propagation neural network to predict the Shanghai Composite Index. The denoising process was executed on the input data to remove noise. This model achieved more accuracy than did a conventional back propagation neural network based on statistical tests such as mean absolute error, root mean-square error, and mean absolute percentage error.

For time series data, such as text, signals, stock prices, and so on, a long short-term memory (LSTM) is superior for learning temporal patterns in deep neural networks (DNNs). A LSTM overcomes a vanishing gradient problem in a recurrent neural network (RNN) to learn long-term dependencies in time series data using memory cells and gates. Attempts have been made to forecast stock prices using this network. Chen et al. [16] tried to predict stock returns in China using an LSTM. They showed that the prediction accuracy improved as the number of inputs increased. Nelson et al. [17] utilized LSTMs to predict future trends in stock prices using stock price and technical analysis indicators. Experimental results showed that their proposed LSTM was more accurate than other machine learning models, such as random forest, multilayer perceptron, and pseudo-random models. Bao et al. [3] used a three-stage process to predict six market index futures. First, they used a wavelet transformation to reduce high dimensionality stock data to low dimensionality signal data. Second, these data were reproduced using a stacked autoencoder. Finally, they used an LSTM to predict stock prices. They confirmed that the performance of the proposed model was better than those of other models, such as RNN, LSTM, and wavelet-LSTM omitted second-stage models.

Financial time series data can be used not only as numeric data but also as image data that is transformed in predicting stock prices. Technical analysis uses chart images to predict stock prices [18, 19, 20, 21]. This method involves finding patterns in chart images using technical indicators such as moving averages, Bollinger bands, stochastic oscillators, and so on [6, 22]. DNNs, especially convolutional neural networks (CNNs), can learn or extract these features themselves. CNNs have been outstanding for deep learning techniques in the computer vision field, object detection, segmentation, and so on. Through the ImageNet challenge, one of the biggest challenges in the computer vision field, many models, such as AlexNet [23] (Krizhevsky et al., 2012), VGGNet [24] (Simonyan and Zisserman, 2014), GoogLeNet [25] (Szegedy et al., 2015), and ResNet [26] (He et al., 2016), have distinguished themselves and are still used frequently. Recent attempts have tried to apply stock chart images to CNNs. Chen et al. [27] used not only CNNs but also data visualization methods, such as Gramian angular fields, moving average mapping, and double moving average mapping, for the purpose of transforming stock price data into image data to eliminate noise. They showed that the proposed model was more accurate than a candlestick chart without the data visualization methods applied. Hu et al. [28] proposed a three-stage investment decision strategy to construct a low-risk and high-return portfolio. First, they used the convolutional autoencoder method as a tool to extract nonlinear features of the candlestick chart. Second, they clustered the features in a hidden weight layer in the autoencoder. Finally, they constructed a portfolio based on the Sharpe ratio from each cluster. The performance of this method was better than that of the Financial Times Stock Exchange 100 Index.

Studies have looked at improving the accuracy of predicting target values by fusing data from different data types or other resources rather than learning from one representation of the data. Ba et al. [29] proposed a deep multimodal neural network using a CNN and a multi-layer perceptron to classify the dataset. They used Caltech-USCD bird and flower data and Wikipedia articles corresponding to the image data, and they extracted features of encyclopedia articles to complement the image data features to predict unseen image classes. The empirical results of this study indicated that their proposed model had a better performance than that of a single model. Ma et al. [30] achieved significant results in matching bidirectional images and sentences using a multimodal CNN with image data and sentence data. Wang et al. [31] used an ensemble CNN with an RNN to classify multi-label images. The RNN was used to learn the features of the label dependency of the image data. This model performed better than did state-of-the-art models, such as K-nearest neighbor search models, softmax prediction models, metric learning models, and so on. Donahue et al. [32] proposed a long-term recurrent convolutional network comprised of a convolutional network and LSTM for a video recognition task. They replaced fully connected layers with LSTM in the CNN to learn sequential vision features. Attempts have been made to construct feature fusion-based forecasting models for financial time series. Guo et al. [33] used three feature selection techniques, specifically, independent component analysis, canonical correlation analysis, and the support vector machine technique, to preprocess raw data and remove noise. The proposed model, which fused three feature selection techniques, had a better performance than those of other models that combined two feature selection techniques. However, this study used only one representation for the financial time series data.

An optimal architecture constructed from different representations will learn duplicate features but will also learn the different characteristics of each architecture, which can improve the prediction accuracy. From this motivation, in this study, we propose a feature fusion model that integrates a CNN and LSTM to fuse features of different representations from financial time series data to improve accuracy in predicting stock prices. We call this proposed model a feature fusion LSTM-CNN model. This model learns the patterns of chart images and reflects the temporal characteristics contained in the financial time series data. Before building a feature fusion LSTM-CNN model, we have to construct each model optimized for this particular data representation. To construct a CNN that is optimized for stock chart images, we use residual learning and bottleneck architecture to extract hidden patterns in the stock chart images [26]. We call this model a stock chart CNN (SC-CNN). Next, we design the optimal LSTM model using temporal information on close prices and trading volumes. We call this model a stock time series LSTM (ST-LSTM). Finally, we construct the feature fusion LSTM-CNN model by combining the SC-CNN and ST-LSTM models by taking the characteristics of both the chart image data and the time series data. We call the proposed model a feature fusion LSTM-CNN model. When we train the feature fusion LSTM-CNN model, we use joint training to reflect each model training procedure simultaneously to improve the efficiency of the proposed model. In this study, we use minute-by-minute SPDR S&P 500 ETF Trust (SPY) ticker data as the financial time series data because it has the largest trading volume among ETF markets. Using this data, we create different representations to fit our models. We create four stock chart images to check which image is the most appropriate for predicting stock prices. Then, using early fusion, we create three fusion chart images that combine stock chart images with volume information to incorporate more information. As inputs of the ST-LSTM model, we use the returns of close prices and trading volume data. Using these representations, we verify that the feature fusion LSTM-CNN model performs better than the single models (SC-CNN and ST-LSTM) do.

The remainder of the paper consists of four sections. Section 2 explains the materials and methods included in our suggested model. Section 3 details our experimental procedure. The experimental results and discussion are described in Section 4. Finally, Section 5 provides our conclusions of this study.

## Materials and methods

### Data representation

In this study, we use minute-by-minute SPY ticker data, which has the largest trading volume among ETF markets. We collect trade high, low, open, and close price and volume data, which cover 97,474 data points running from October 14, 2016 to October 16, 2017, from the Thomson Reuter Database. Using this financial time series data, we create different representations to match the input types of each model to extract features from the CNN and LSTM.

**Fig 1:**
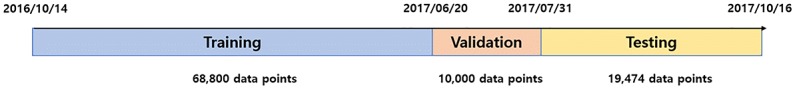
Setting training, validation and testing dataset during the whole sample period.

Fig 1 shows how we set the training, validation, and testing dataset. We use the 68,800 data points from October 14, 2016, to June 20, 2017, as training data; 10,000 data points from June 21, 2017, to July 31, 2017 as validation data; and 19,474 data points until October 16, 2017, as testing data.

**Fig 2:**
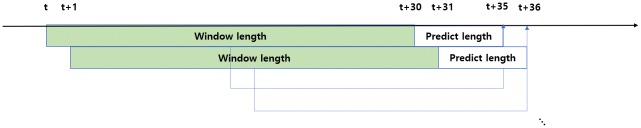
Setting window length, predict length and rolling window during the whole sample period.

In Fig 2, we set the window length to 30 minutes, rolling window to 1 minute, and predict term to 5 minutes. It means we will predict the stock price after five minutes by looking at the data for the previous 30 minutes based on a minute-by-minute current time point. These settings are applicable for each representation since we combine the CNN with LSTM.

#### Stock chart images

**Fig 3:**
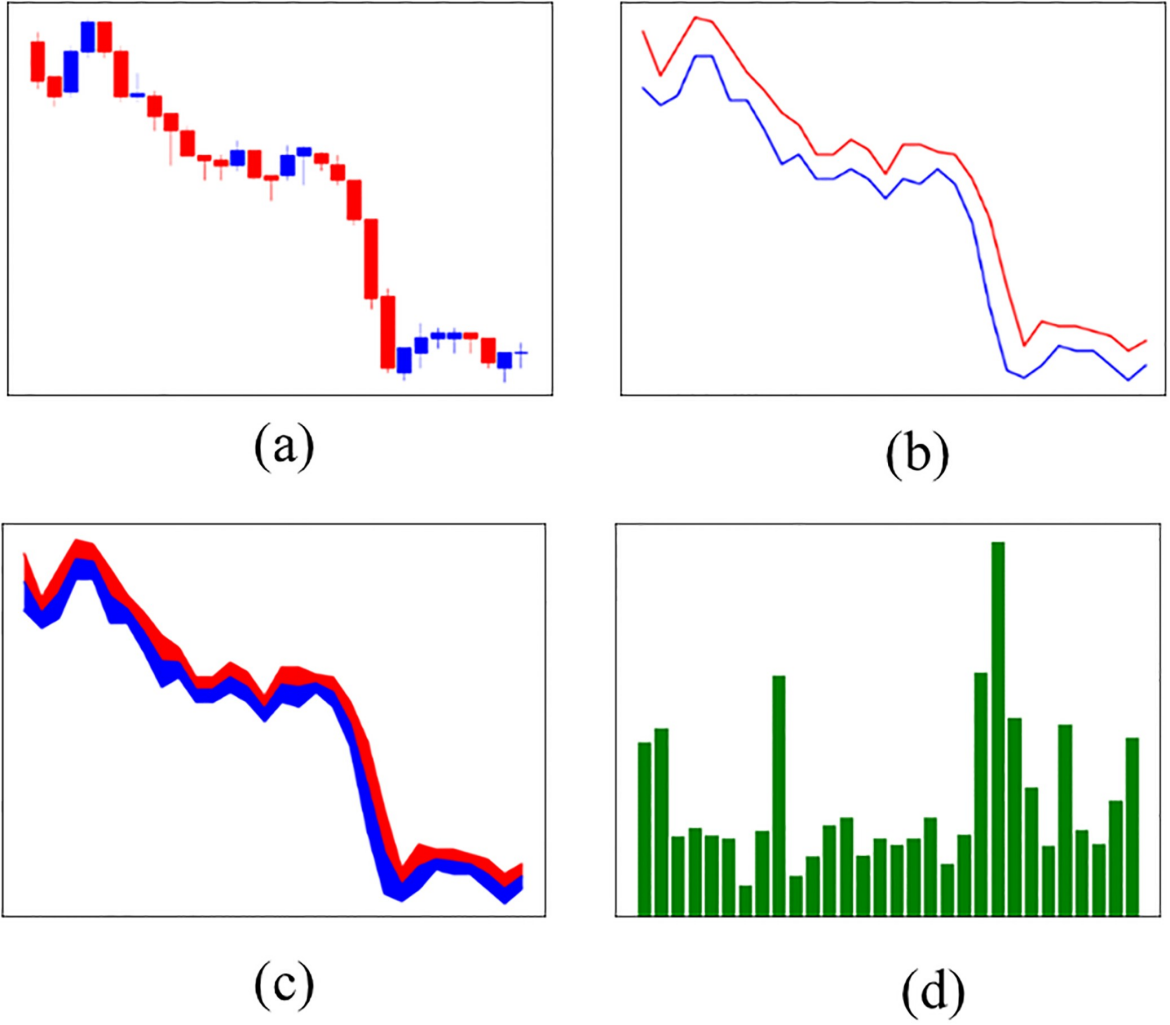
Four stock chart images using SPY data. The time interval is between *t* − 30 and *t*. (a) Candlestick chart, (b) Line chart, (c) F-line chart, and (d) Bar chart.

Using the financial time series data, we create four stock chart images as inputs for the CNN, as shown in Fig 3. All of the stock chart images use RGB colors. Fig 3a is a candlestick chart that is comprised of high, low, open, and close prices. Candlestick charts have often been used to identify patterns [34–36]. Fig 3b is a line chart that is comprised of high and low prices. Siripurapu [37] tried to predict stock prices using a line chart, but the experiment failed because of the lack of information in the chart image. To create the model in this experiment, we incorporate a middle price by averaging the high and low prices, and we then fill the colors between the prices to provide more information to the CNN. We call this chart a filled line chart (f-line chart), which is shown in Fig 3c. In addition to stock price data, trading volume data plays an important role in predicting stock prices [5, 38]. Based on this notion, we construct bar charts of the trading volume data to determine whether this data, reconstructed as an image, serves as a key feature to predict stock prices. Fig 3d is a bar chart of trading volume data. To input the data into the CNN, we resize and crop these images to be 112x112 pixels.

#### Fusion chart images

**Fig 4:**
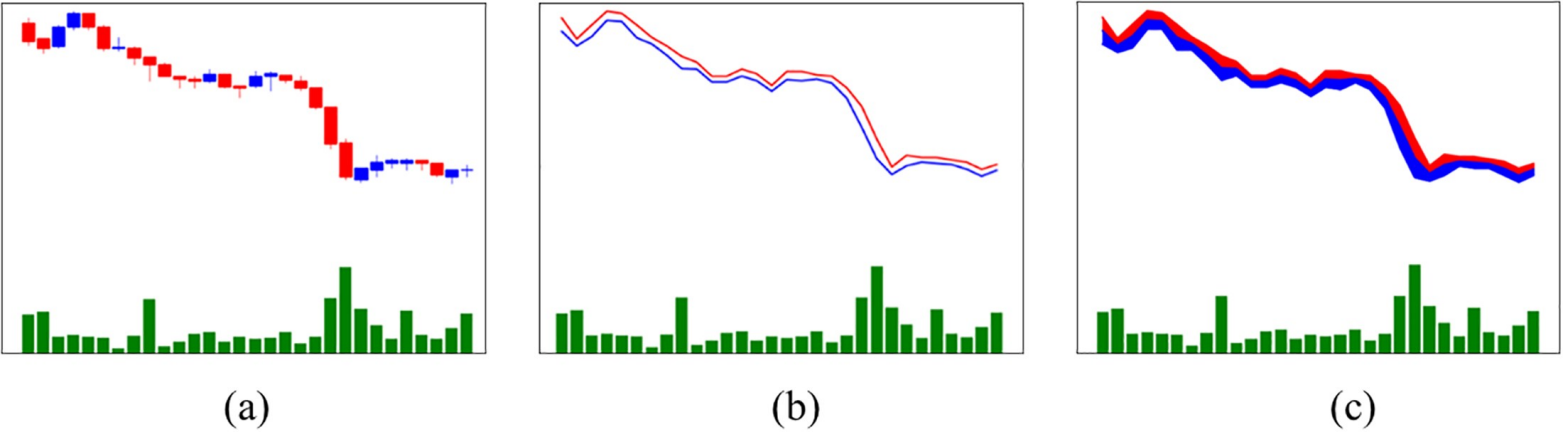
Three fusion chart images using early fusion. The time interval is between *t* − 30 and t. (a) Candlebar chart, (b) Linebar chart, and (c) F-linebar chart.

In the case of supervised learning, each piece of data should have a corresponding label. However, similar data, such as video data, multi-label image data, and so on, may correspond to one label. By extracting the features of these data and expressing them as one data type, the amount of information can be increased, making learning more efficient. Li et al. [39] described an image fusion method using a wavelet transform-based approach. They showed that this method provided significant results when using image fusion. Snoek et al. [40] compared the performance of early fusion with that of late fusion methods. Early fusion methods are used to combine components of video data, such as visual features, auditory features, and textual features, before executing a trading algorithm. In contrast, late fusion methods combine these input features after executing trading algorithms separately. Snoek et al. [37] also defined early fusion that integrates unimodal features before learning concepts. In this study, we will fuse stock charts with the bar chart shown in Fig 3 using the early fusion method since the bar chart is used as an important factor in sharing the label with the stock charts to predict stock prices. We call the resulting image a fusion chart image. We create three fusion chart images that combine the stock price charts (i.e., the candlestick chart, line chart, f-line chart, and bar chart) to check whether or not the fusion chart images perform better. Fig 4 shows three fusion chart images using the early fusion method. Fig 4a is a combination of a candlestick chart and a bar chart that we call a candlebar chart. Fig 4b is a combination of a line chart and bar chart that we call a linebar chart. Fig 4c is a combination of an f-line chart and bar chart that we call an f-linebar chart. The sizes of these images are the same as those of the stock chart images, as described in section 2.1.1.

#### Stock time series data

**Fig 5:**
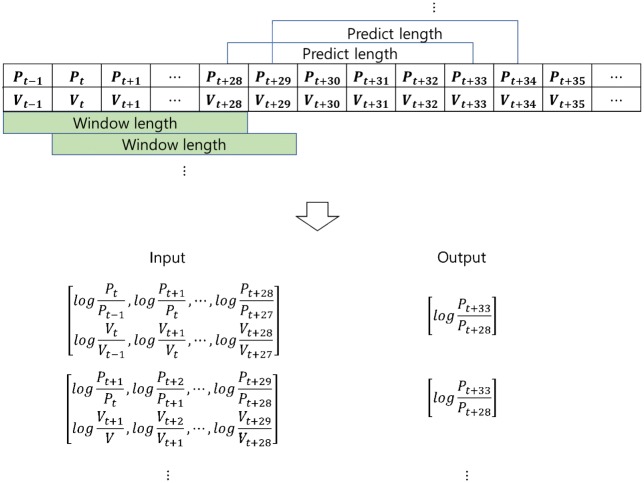
Preprocessing stock time series data using logarithmic return based on the window length and predict length.

The data used in the LSTM should be time series data to extract the sequential features of the data. In this study, we choose adjusted close price and trading volume data as inputs to the LSTM. Fig 5 shows the preprocess of stock time series data.

As making an input data, we take adjusted close price and volume data based on window length, its dimension could be 30x2. We transformed this dataset into logarithmic returns to reduce noise using (Eq 1). Dimensions of the data is 29x2. As making an output data, since our goal is to predict stock prices, we take adjusted close price only. Based on the last sequence of the input data, we take this value and adjusted close price which takes the predict length into account. For example, in Fig 5, we have sequences of adjusted close price and volume data. Based on window length, we transformed *P*_*t−*1_, *P*_*t*_, ⋯, *P*_*t*+28_ and *V*_*t−*1_, *V*_*t*_, ⋯, *V*_*t*+28_ into $log\frac{P_{t}}{P_{t-1}},log\frac{P_{t+1}}{P_{t}},\cdots ,log\frac{P_{t+28}}{P_{t+27}}$ and $log\frac{V_{t}}{V_{t-1}},log\frac{V_{t+1}}{V_{t}},\cdots ,log\frac{V_{t+28}}{V_{t+27}}$ where *P*_*t−*1_, *V*_*t−*1_ are stock price and trading volume at time *t* − 1 using (Eq 1). The corresponding value of output is $log\frac{P_{t+33}}{P_{t+28}}$ which is based on five minutes predict length. We will apply these output data equally to the output of the chart image because the data representations are made from different but identical input data.

$$Logarithmic\;return\;:\;R_{t}\;=\;log\frac{P_{t}}{P_{t-1}}$$ (1)

where *P*_*t−*1_ and *P*_*t*_ are the prices at times *t* − 1 and *t*, respectively, and R_*t*_ is the logarithmic return.

### Performance measure

We select three performance measures, the root mean square error (RMSE), the root mean absolute error (RMAE), and the MAPE, to evaluate the predictive power of our proposed models. When we train models, we use RMSE as a loss function, which means that the model is trained to reduce RMSE. RMSE is a good measure for revealing relatively large forecast errors [41], RMAE is useful for revealing the systematic bias of the model, and MAPE is a measure of the accuracy of predictions in statistics. These equations are as follows:

$$RMSE\;=\;\sqrt{\frac{1}{N}\sum_{i\;=\;1}^{N}{\left(x_{1,i}-x_{2,i}\right)}^{2}}$$ (2)

$$RMAE\;=\;\sqrt{\frac{1}{N}\sum_{i\;=\;1}^{N}\left|x_{1,i}-x_{2,i}\right|}$$ (3)

$$MAPE\;=\;\frac{100}{N}\sum_{i\;=\;1}^{N}\left|\frac{x_{2,i}-x_{1,i}}{x_{1,i}}\right|$$ (4)

where *N* is the number of data points, *x*_1,*i*_ is a predicted value, and *x*_2,*i*_ is a real value.

### Stock chart CNN

The performance of the network can be improved by deepening the network. This method has complicated feature representation capacity by using a complex function that increases the non-linearity in extracting features, improving the performance of the network. However, due to the network deepening, not only can overfitting occur because of the vanishing gradient problem but also a degradation problem, through which the training loss increases despite the deepening of the network, may arise [26]. Therefore, using a method to prevent overfitting and the degradation problem while keeping the network deep becomes important. ResNet has overcome this problem by using residual learning and bottleneck methods [26]. We will use these methods to create a CNN that is optimized for stock chart images.

#### Residual learning and bottleneck structure

**Fig 6:**
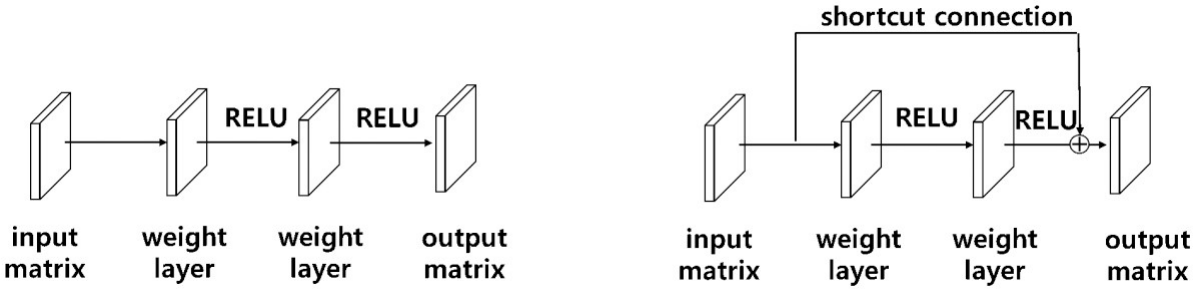
Residual learning. (a) Stacked layers (b) Stacked layers using a shortcut connection.

Fig 6a shows a common method to extract features of input *X* by passing through existing weight layers.

A degradation problem may occur even if the network is deeply piled up. To solve this problem, He et al. [26] used a shortcut connection for residual learning, as shown in Fig 6b. In the case of a shortcut connection, the input *X* is mapped to the feature *F*(*X*) through the activation function without going through the weight layer.

F(X)≔H(X)-X (5)

Using this shortcut connection, *H*(*X*), or *F*(*X*) − *X* rather than *F*(*X*), is treated as an optimized mapping by assuming (Eq 5), as follows, where *X* is an input matrix and *F*(*X*) and *H*(*X*) are output matrices. Setting the residual to zero makes the optimization easier. This method can solve the problem of degradation due to the deepening of the network [26].

**Fig 7:**
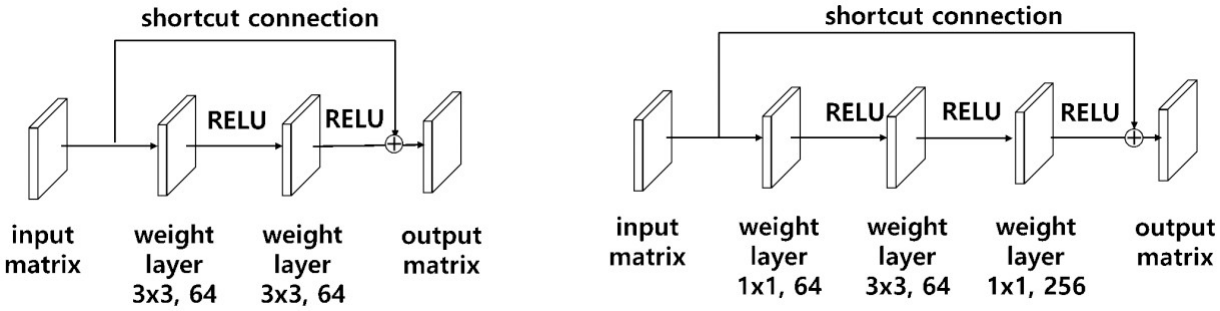
Bottleneck architecture. (a) A building block (b) A bottleneck building block.

Fig 7b represents a bottleneck structure that is designed with three weight layers. The feature of this structure is that it decreases time complexity by reducing the computed parameters and increases the number of filters about four times, which can extract many features, by reconstructing 3x3 64 filters as 1x1 64 filters, 3x3 64 filters, and 1x1 256 filters, which can be compared to Fig 7a. Thus, this structure can be given a substantial amount of information.

#### Proposed SC-CNN model

**Fig 8:**
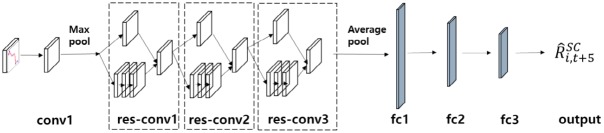
Construction of the SC-CNN model using residual learning and the bottleneck method.

A variety of networks use residual learning and bottleneck architecture [42–44]. Using these methods, we construct the proposed SC-CNN model, which is optimized for the stock. Fig 8 represents our proposed SC-CNN model.

In Fig 8, conv1 is a convolutional layer; res-conv1, res-conv2, and res-conv3 are constructed using the residual learning and bottleneck methods; and the fully connected layers are abbreviated as fc1, fc2, and fc3.

We modify the ResNet-50 model to match our stock chart images. In the input stage, we resize and crop the stock chart images so that they are 112x112 pixels, and we then fine-tune the numbers of convolutional and fully connected layers as well as hyperparameters, such as the dimension of convolutional layers, the number of neurons in fully connected layers, the dropout ratio, and so on, using trial and error. The SC-CNN model is not quite as deep as the ResNet-50 model, which has fifty layers, because stock chart images are low-dimensional data. However, in order to extract the nonlinear characteristics of the chart images, we try to maintain the depth by using these methods. From this procedure, we construct four convolution layers and three fully connected layers. Table 1 shows the architectures of the SC-CNN model optimized for stock chart images as a result of trial and error. The experimental procedure will be described in section 3.

**Table 1:** Architectures of the SC-CNN model.

Layer name	Output size	Kernel size	Pad	Stride	Dropout
**conv1**	32x56x56	7x7	3	2	
**max pool**	32x28x28	3x3	2		
**res-conv1**	128x28x28	1x1	0	1	
		3x3	1	1	
		1x1	0	1	
**res-conv2**	256x14x14	1x1	0	2	
		3x3	1	1	
		1x1	0	1	
**res-conv3**	512x7x7	1x1	0	2	
		3x3	1	1	
		1x1	0	1	
**average pool**	512x1x1	7x7	0		
**fc1**	500				0.5
**fc2**	100				0.5
**fc3**	25				0.5

### Stock time-series LSTM

#### Long short-term memory

**Fig 9:**
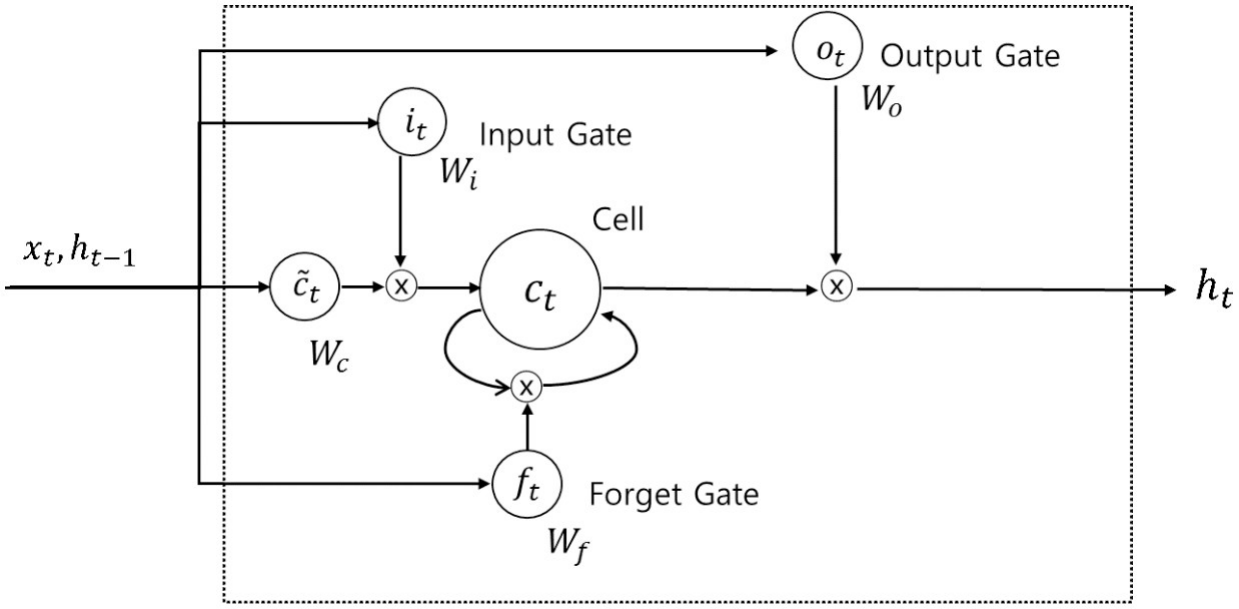
Memory block of LSTM.

In addition to image data, various other data types are used in applying deep learning technology; in particular, financial data are often time-series data. RNNs were initially used to learn the sequential patterns of time series data. However, in the case of RNNs, the problem of the vanishing gradient, which occurs as the network deepens, has not been solved. The network that solved this problem was LSTM. Hochreiter and Schmidhuber [45] used gate process and memory blocks to solve the vanishing gradient problems in the RNN context. Fig 9 shows the memory block of an LSTM, and Eqs (6) to (11) show the calculations for each gate and cell state,

$$f_{t}\;=\;\sigma (W_{xf}x_{t}+W_{hf}h_{t-1}+b_{f})$$ (6)

$$i_{t}\;=\;\sigma (W_{xi}x_{t}+W_{hi}h_{t-1}+b_{i})$$ (7)

$${\tilde{c}}_{t}\;=\;tanh(W_{xc}x_{t}+W_{hc}h_{t-1}+b_{c})$$ (8)

$$c_{t}\;=\;f_{t}*c_{t-1}+\;i_{t}*{\tilde{c}}_{t}$$ (9)

$$o_{t}\;=\;\sigma (W_{xo}x_{t}+W_{ho}h_{t-1}+b_{o})$$ (10)

$$h_{t}\;=\;o_{t}*tanh(c_{t})$$ (11)

where *W* represents weight matrices, *b* is a bias term, *σ*(∙) is a sigmoid function, and *tanh*(∙) is a hyperbolic tangent function. In Fig 6, the input variables *x*_*t*_, and *h*_*t*−1_ go into the four gates labelled as *f*_*t*_, *i*_*t*_, *o*_*t*_, ${\tilde{c}}_{t}$. For the input and output gates, the weights corresponding to each gate are calculated, and the sigmoid function is taken as the activation function. The sigmoid function takes a value between zero and one. If the output value is one, the corresponding value should be kept, but if it zero, the corresponding value should be completely discarded. For the remaining gate, the input modulate gate, *tanh* is used to determine how much new information should be reflected in the cell state. Finally, the information to be reflected in *c*_*t*_ is calculated by adding the point-wise multiplication of the previously calculated *i*_*t*_, ${\tilde{c}}_{t}$ values and the values calculated from the forget gate, the previous cell state value, and the point-wise multiplication of *c*_*t*_. Finally, in order to calculate the output value *h*_*t*_, point-wise multiplication is performed on the value calculated from the output gate and the value that is obtained by adding *tanh*(∙) to the calculated cell state value.

#### Proposed ST-LSTM model

**Fig 10:**
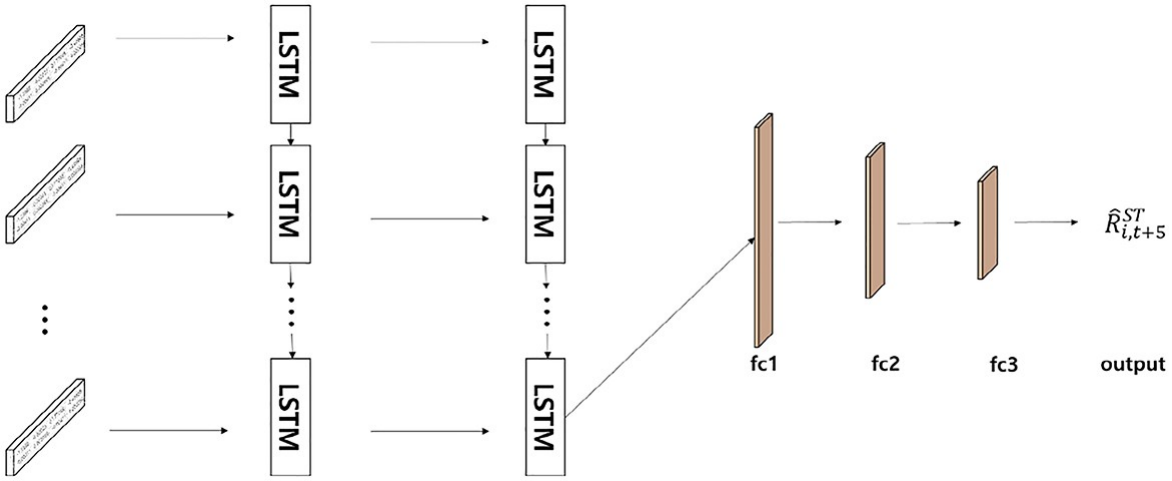
The architecture of the ST-LSTM model.

In order to extract the sequential features of the stock time-series data, we design the optimal LSTM model. Fig 10 shows our proposed ST-LSTM model for stock time-series data. As mentioned in section 2.1.3 above, we use close price and volume data, which is transformed to logarithmic returns, as input data to maintain relationships with the fusion chart images that reconstruct stock price and trading volume data as image data.

The data are generated as a stacked data type so that the data can be inputted simultaneously. The model is designed to predict the value at time *t* + 5 using values from time *t* − 30 to *t*, as in the case of the stock chart data. The structure of the model consists of two LSTM layers and three fully connected layers determined by trial and error. Fully connected layers are configured to improve the nonlinear prediction power by constructing three layers and to fuse this model with the features extracted from the chart data in our feature fusion LSTM-CNN model.

### Proposed feature fusion LSTM-CNN model

**Fig 11:**
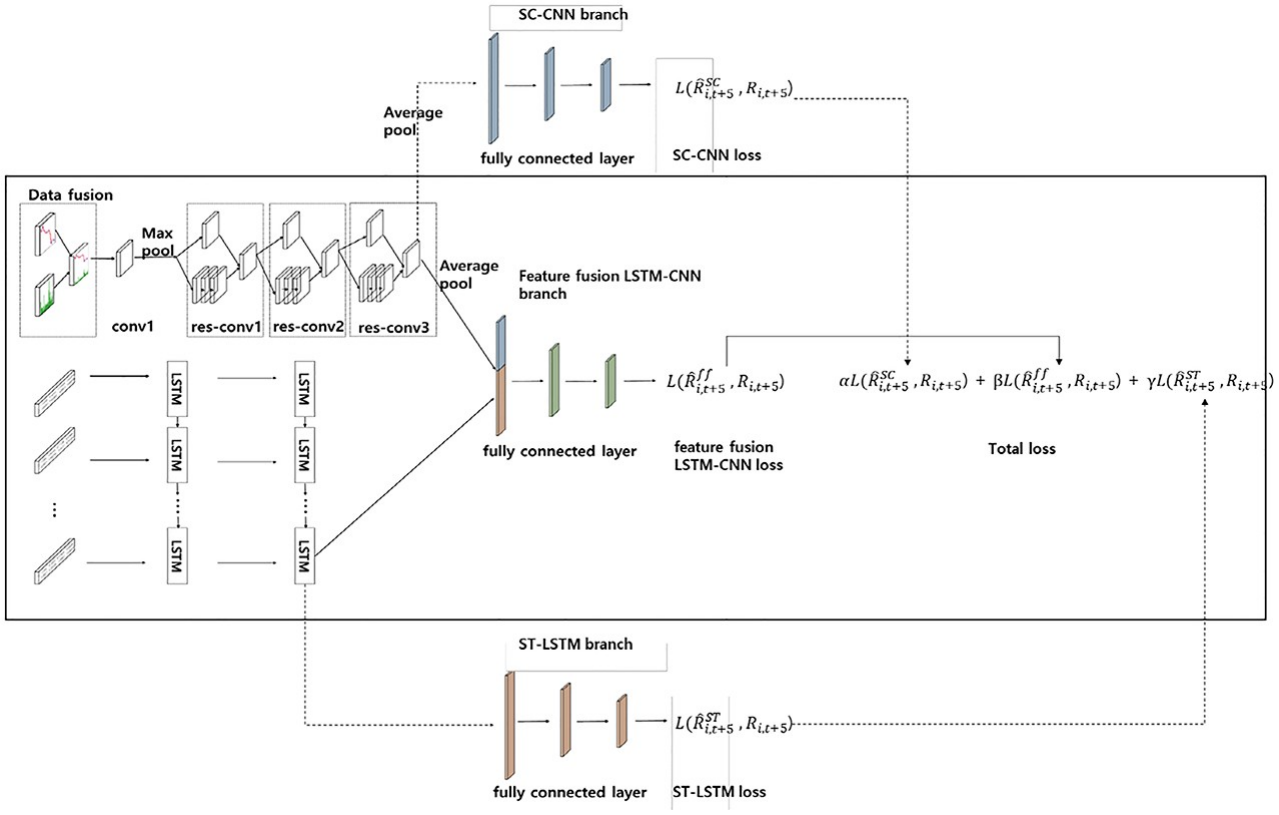
The architecture of proposed feature fusion LSTM-CNN model comprised of SC-CNN and ST-LSTM models.

In predicting stock prices, the fusion of the different features comprising the extracted stock chart images and stock time series data from the same data, can improve the training model. Fig 11 represents the architecture of our proposed feature fusion LSTM-CNN model, whose construction is carried out through a total of three steps. The first stage takes the optimal architectures of the SC-CNN model. In this case, the SC-CNN branch is not included as only part of the convolution layers is included. We use the same architecture as in Table 1, which is based on our experiments with the stock chart images shown in Fig 3 using the SC-CNN model. The second step is developing the ST-LSTM model. In this case, the ST-LSTM branch is also not included as only part of the LSTM layers is included. The final step of developing the proposed model involves fusing the graphical features of the SC-CNN model and temporal features of ST-LSTM model at the fully connected layer. We called this fully connected layer as the feature fusion LSTM-CNN branch.

In training our proposed model, we applied the joint-training method, wherein we can achieve upper-bound performances of our proposed method [46, 47]. The loss functions of the models are defined as follows in Eqs (12) to (15):

$$L({\hat{R}}_{i,t+5}^{SC},\;R_{i,t+5})\;=\;\sqrt{\frac{1}{N}\sum_{i\;=\;1}^{N}{\left({\hat{R}}_{i,t+5}^{sc}-R_{i,t+5}\right)}^{2}}$$ (12)

$$L({\hat{R}}_{i,t+5}^{FF},\;R_{i,t+5})\;=\;\sqrt{\frac{1}{N}\sum_{i\;=\;1}^{N}{\left({\hat{R}}_{i,t+5}^{ff}-R_{i,t+5}\right)}^{2}}$$ (13)

$$L({\hat{R}}_{i,t+5}^{ST},\;R_{i,t+5})\;=\;\sqrt{\frac{1}{N}\sum_{i\;=\;1}^{N}{\left({\hat{R}}_{i,t+5}^{st}-R_{i,t+5}\right)}^{2}}$$ (14)

$$Total\;RMSE\;=\;\alpha *L({\hat{R}}_{i,t+5}^{SC},\;R_{i,t+5})+\;\beta *L({\hat{R}}_{i,t+5}^{FF},\;R_{i,t+5})+\;\gamma *L({\hat{R}}_{i,t+5}^{ST},\;R_{i,t+5})$$ (15)

where *N* is the number of data points, $\hat{R}$ is a predicted return value, and *R* is a target return value. $L({\hat{R}}_{i,t+5}^{SC},\;R_{i,t+5})$ is the loss function of the SC-CNN model, $L({\hat{R}}_{i,t+5}^{FF},\;R_{i,t+5})$ is the loss function of the feature fusion LSTM-CNN model, and $L({\hat{R}}_{i,t+5}^{ST},\;R_{i,t+5})$ is the loss function of the ST-LSTM model. The values of *α*, *β*, and *γ* are the weights of the total RMSE. These parameters indicate the degree of reflection of each model loss.

We take not only the fusion of image and temporal features, which come from concatenating each feature, but also each separate attribute such as image and temporal features. It is composed of three steps. First, SC-CNN is trained to reduce SC-CNN loss in (Eq 12). This process involves learning the graphical features of the chart image. Second, ST-LSTM is trained to reduce ST-LSTM loss in (Eq 14). In this process, the temporal feature is learned from the stock time series. Finally, feature fusion LSTM-CNN is trained to reduce feature fusion LSTM-CNN loss in (Eq 13). In this process, feature fusion LSTM-CNN shares the parameters that are contained in convolutional layer in SC-CNN and LSTM layer in ST-LSTM. However, the SC-CNN, ST-LSTM, and feature fusion LSTM-CNN branches are independent. From these three losses, we can derive the total RMSE using (Eq 15). We set β so that the feature fusion LSTM-CNN loss is reflected more than the other loss values. For example, we set the hyperparameters α, β, and γ to 0.2, 1, and 0.2, respectively, to reflect the feature fusion LSTM-CNN loss to be more than the two other losses. By taking total RMSE, feature fusion LSTM-CNN can be trained for various features. In addition, a regularization effect can also be expected to prevent overfitting of features of the fused feature by taking SC-CNN and ST-LSTM losses.

## Experiment

We proceed with the experiment as follows. First, we construct the optimized stock chart images for the SC-CNN model shown in Fig 3, and we then check which stock chart image is the most appropriate for predicting stock prices. Second, we use the fusion chart images shown in Fig 4 to check if these images have better performances than those of stock chart images that are not fused using the SC-CNN model. The result of this experiment shows that adding information to the chart image is meaningful in predicting stock prices. Then, we create the ST-LSTM model that includes time series information to build our feature fusion LSTM-CNN model. In this case, we construct the optimal ST-LSTM model by taking 29x2 dimensional logarithmic returns of close price and volume data from time *t* − 30 to time *t*. Finally, we describe the fusion of different representation features of the same data by constructing the feature fusion LSTM-CNN model that we are proposing in this study. Here, we explain how to fuse and train the SC-CNN model to extract features using chart image data and the ST-LSTM model to extract features from financial time series data.

Unlike the image data used in the ImageNet challenge, our chart image data come from low-dimensional datasets and do not require as deep a CNN. If we use the ResNet architecture without modification, overfitting is more likely to occur. Reflecting this problem, we use residual learning and the bottleneck method to capture the nonlinear features of the data by increasing the depth of the network. Furthermore, we construct three fully connected layers to improve the nonlinear predictive power. The dimensions of the convolutional layers and the outputs of the fully connected layers are determined by trial and error, and these values are shown in Table 1. In addition, we add dropout effects to the fully connected layers of 0.5 to mitigate the overfitting problem [48], as we use the residual learning and bottleneck methods. In Fig 3, the input data are stock chart images, and we fine-tune the hyperparameters to identify the features of the chart images. We set the learning rate, weight decay, and iteration to 0.001, 0.0005, and 60,000 trials, respectively. From this experiment, we can construct an SC-CNN model that is optimized for stock chart images and find the optimal stock chart images for predicting stock prices.

From the previous experiments, we obtain a SC-CNN model that is suitable for stock chart images. Using this model, we can check whether fusion chart images are better than stock chart images for predicting stock prices. Using the early fusion method, fusion chart images are created by fusing bar charts with other stock chart images, as shown in Fig 4. The architecture of the SC-CNN model and the hyperparameters, such as the learning ratio, weight decay, number of iterations, and so on, are the same as in past experiments since they are already optimized for stock chart images. Furthermore, these settings increase the reliability of our experiment without changing the parameters. From this experiment, we can check whether fusion chart images are better than stock chart images.

In the previous two experiments, chart image data are used as input data. In addition to the features that can be extracted from the chart images in estimating stock prices, since stock prices are a time series, this sequential information can help to predict stock prices if the network is constructed using fusion with the features extracted from the images. In this experiment, we use stock time series data, specifically, close price and trading volume data. In forecasting stock prices, the close price has been used in the literature as an input [4, 6, 11, 15, 49] and trading volumes are also an important factor in predicting stock prices [5, 38]. We use the log returns of the close price and trading volume data instead of the raw data to remove noise. We also stack the data in a 29x2 form to simultaneously input these two values. Using these input data, we construct two stacked LSTM layers and three fully connected layers. The outputs of the LSTM layers and fully connected layers are decided by trial and error. Hyperparameters such as batch size, learning rate, and weight decay are set equal to their values in the above experiments. From this experiment, we can check which features are better for predicting stock prices. Based on this result, when we fuse different features from each model, we can determine the extent to which we will reflect each feature in the fully connected layer stage.

We construct SC-CNN and ST-LSTM models that are suitable for stock chart images and stock time series data. Using these two different representations made from the same data, we can extract the corresponding features of each model. By fusing these features, if we train the feature fusion LSTM-CNN model, the difference between the prediction and target values can be reduced. In addition, we adjust the joint training by reflecting the losses of the SC-CNN and ST-LSTM models simultaneously when we train the feature fusion LSTM-CNN model. Doing so can improve the performance of the feature fusion LSTM-CNN model. Fig 11 shows our proposed model. The training procedure is composed of three stages. In the input phase, the chart image and time series data, which share the same time interval, enter the SC-CNN model and the ST-LSTM model, which are made up of the same data in the input phase, respectively. In the first stage, the SC-CNN model is trained in the same way as in the previous experiment. The architectures of the SC-CNN model used in the feature fusion LSTM-CNN model are the same as those described in Table 1. The second stage is to train the ST-LSTM model using stock time series data. This architecture is the same as that described in section 2.4.2. These procedures are part of the joint training to increase the efficiency of the feature fusion LSTM-CNN model. The last stage is to fuse the features of the different representations from each model. The fusion of these features is generated in the first fully connected layer. We determine to what extent the features of each model are reflected in the feature fusion LSTM-CNN model. These three stages of the training process are executed together.

When we test feature fusion LSTM-CNN, we do not use the SC-CNN and ST-LSTM branches because these are used to train various features. In addition, these branches prevent overfitting of features of the fused feature like regularization. It is not used when we predict stock price. Therefore, we only use convolutional layers, LSTM layers and feature fusion LSTM-CNN branch when we test our out of sample dataset. We draw connection of convolution layer with SC-CNN branch and LSTM layer with ST-LSTM branch to express when we test out of sample dataset, these branches are removed.

## Results and discussion

We check three results of this experiment. First, we determine the optimal stock chart image to predict stock prices. Second, we show that fusion chart images perform better than stock chart images do. Last, we need to make sure that our proposed feature fusion LSTM-CNN model is meaningful. As we mentioned in section 2.2, we use RMSE, RMAE, and MAPE as performance measures.

We create four stock chart images using financial time series data to find the optimal stock chart image to predict stock prices. Table 2 shows the out-of-sample results of an experiment with stock chart images using the SC-CNN model.

**Table 2:** Comparison of out-of-sample results for stock chart images using the SC-CNN model.

Data	RMSE	RMAE	MAPE
**Candlestick**	0.1258	0.2896	0.0338
**Line**	0.1800	0.3756	0.0568
**F-line**	0.1549	0.3427	0.0473
**Bar**	0.1278	0.2933	0.0347

The out-of-sample results were judged based on the RMSE, RMAE, and MAPE. The test results of candlestick chart were 0.1258 (RMSE), 0.2896 (RMAE), and 0.0338 (MAPE), which are the lowest values among four stock chart images. Unlike line and f-line charts, candlestick charts contain more information because the open, high, low, and close information are all represented separately. In the case of the bar chart, the test results were 0.1278 (RMSE), 0.2933 (RMAE), 0.0347 (MAPE), which are the second lowest among the stock chart images. It can be confirmed that the reconstruction of volume information also serves as important raw data for stock predictions [5, 38]. Furthermore, adding information to the chart image can be significant for predicting stock prices based on the f-line chart and line chart results. The f-line chart results were 13.95% (RMSE), 8.75% (RMAE), 16.70% (MAPE), which are better than those of the line chart. Thus, the performance of the model can be improved by adding information to the input data. This result can be extended to fusion chart images since these images contain more information than do stock chart images that are not yet fused.

Next, we check whether fusion chart images are more appropriate for predicting stock prices as compared to stock chart images. Table 3 shows the out-of-sample results for fusion chart images using the SC-CNN model based on the RMSE, RMAE, and MAPE.

**Table 3:** Comparison of out-of-sample results for fusion chart images using the SC-CNN model.

Data	RMSE	RMAE	MAPE
**Candlebar**	0.1241	0.2862	0.0330
**Linebar**	0.1685	0.3727	0.0559
**F-linebar**	0.1436	0.3287	0.0435

Compared with the stock chart images, the test results for the candlebar chart show 1.33%, 1.19%, and 2.35% improvements in terms of the RMSE, RMAE, and MAPE, respectively. The linebar chart shows improvements of 6.35% (RMSE), 7.77% (RMAE), and 1.59% (MAPE) as compared to the line chart. Finally, the f-linebar chart also shows improvements of 7.24% (RMSE), 4.09% (RMAE), and 8.00% (MAPE) in comparison with the f-line chart. All of the fusion chart images have better performances than those of the stock chart images that do not incorporate fusion. This result shows that it is efficient to add more information to stock chart images. Although the candlebar chart does not show as large of an improvement as the other fusion charts do, it is the most appropriate of the chart images. Therefore, when we construct the feature fusion LSTM-CNN model, we will only use fusion chart images.

Table 4 represents the out-of-sample results of the ST-LSTM model using stock time series data.

**Table 4:** Comparison of out-of-sample results for stock time series data using the ST-LSTM model.

Data	RMSE	RMAE	MAPE
**Stock time series**	0.1198	0.2779	0.0311

This model has a better performance than those of the SC-CNN models using fusion chart images and stock chart images. The test results of the ST-LSTM model are 3.49% (RMSE), 2.89% (RMAE), and 5.67% (MAPE) better than those of the candlebar chart, which exhibits the best performance in the SC-CNN model. Thus, the temporal feature, which is included in the stock time series data, is more important than the image data feature for predicting stock prices. This result implies that increasing the proportion of temporal features when feature fusion occurs will be more helpful in predicting stock prices. Therefore, we construct a proposed model to reflect this information by taking more information from the fully connected layer neurons of the ST-LSTM model. We set the temporal feature proportion to 0.6 and the image feature proportion to 0.4 when we construct the feature fusion LSTM-CNN model.

Finally, we should confirm that our proposed model is excellent at predicting stock prices compared to the SC-CNN and ST-LSTM models alone. From previous results, we can determine that the ST-LSTM model is more powerful in predicting stock prices than SC-CNN model is. Thus, we check whether the feature fusion LSTM-CNN model has a better performance than the ST-LSTM model has. Table 5 shows the performance of the feature fusion LSTM-CNN model.

**Table 5:** Comparison of out-of-sample results for stock chart images and stock time series data using the feature fusion LSTM-CNN model.

Data	RMSE	RMAE	MAPE
**Candlebar and stock time series**	0.0980	0.2291	0.0209
**Linebar and stock time series**	0.1081	0.2354	0.0231
**F-linebar and stock time series**	0.1063	0.2318	0.0222

Compared with the results of the ST-LSTM model, the out-of-sample loss of the feature fusion LSTM-CNN model using candlebar charts and stock time series as inputs decreased by 18.18% (RMSE), 17.56% (RMAE), and 32.87% (MAPE). Experiments with other fusion chart images and stock time series showed better performances than that of the ST-LSTM model.

**Fig 12:**
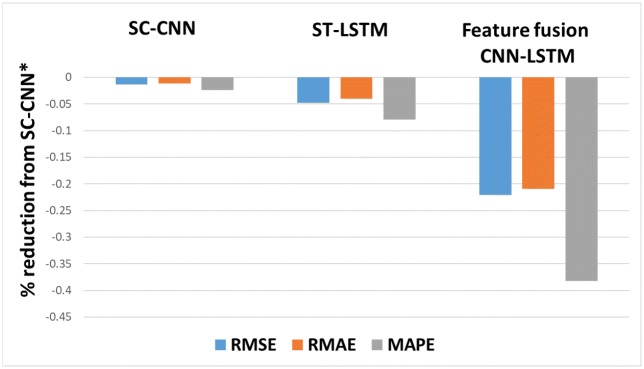
Comparison of prediction errors among three models based on SC-CNN*. The input data are candlebar charts, which are the best performing fusion chart images, and stock time series data. Note: SC-CNN* uses a candlestick chart, which is a stock chart image only.

Fig 12 represents the prediction errors of our three models in comparison with the SC-CNN model, which uses stock chart images as an input. The results show that the feature fusion LSTM-CNN model is the best model for forecasting stock prices.

**Fig 13:**
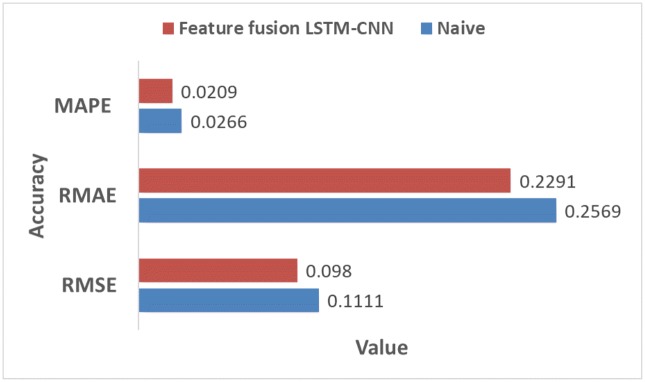
Comparison of accuracy between feature fusion LSTM-CNN and naive model.

We compared the accuracy with the naive model and feature fusion LSTM-CNN model. Naive model is based on the assumption that the value at the previous time point is the same as the value at the later time point. We found that feature fusion LSTM-CNN using fusion chart images and stock time series has outperform the Naive model. Fig 13 shows that comparison of accuracy between naive and feature fusion LSTM-CNN model which use candlebar and stock time series since these data showed the best performance.

The data used here is the adjusted close price of the stock time series data. Compared with naive model, the out of sample results for the feature fusion LSTM-CNN using candlebar and stock time series show 11.79%, 10.82%, and 2.14% improvements in terms of the RMSE, RMAE, and MAPE, respectively. We confirm that our proposed model, feature fusion LSTM-CNN has more accuracy than naive model.

Table 6 shows the results of trading strategies based on five different models. We do not consider trading cost in this experiment.

**Table 6:** Comparison of out-of-sample profit of trading strategies based on five different models.

Data	Model	Profit
**Stock time series**	Naive	0.1548
**Stock time series**	Buy and Hold	0.0309
**Candle**	SC-CNN	0.1559
**Candlebar**	SC-CNN	0.1589
**Stock time series**	ST-LSTM	0.1631
**Candlebar and stock time series**	Feature fusion LSTM-CNN	0.1738

In the case of Naive model, since it is judged that *P*_*t*_ is the same at the time point *t* + 5, it cannot be determined whether it will rise at the time point *t* + 5. Therefore, we assumed trading at each time point and as a result we recorded 15.48% revenue. Buy and hold strategy is a simple trading method to buy shares at the time of starting trading and sell shares at the end of the trading period. We confirmed that revenue was 3.09% when we implemented this strategy. Since our model is constructed to forecast stock price, we can predict stock price at the time point t+5 if we are at the time point t. Therefore, if predicted stock price is above present stock price, we can earn a profit by buying stocks at time point t and selling stocks at the time point t+5. From this point of view, we execute trading simulation in out-of-sample dataset. The results of trading profit using SC-CNN based on candlestick and candlebar chart report 15.59% and 15.89%. Also, ST-LSTM reported 16.31% trading profit. When trading with single models, we can see that the profitability is higher than when the strategy is implemented with buy and hold strategy and naive model. Finally, when we use the feature fusion LSTM-CNN model, we can confirm that it records 17.38% profits which is the highest among all strategies.

**Fig 14:**
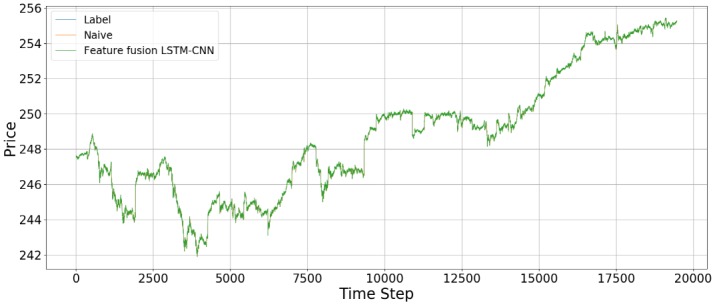
An example of predicting stock prices using the feature fusion LSTM-CNN model and Naive model on the testing dataset. The input data are candlebar chart and stock time series.

**Fig 15:**
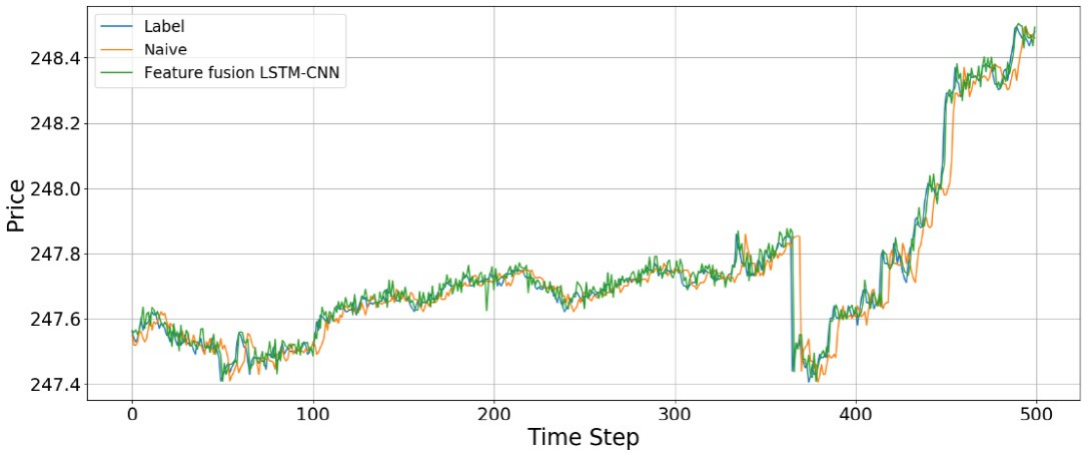
An example of predicting stock prices using the feature fusion LSTM-CNN model with a test dataset of between 0 and 500 data points. The input data are candlebar chart and stock time series.

**Fig 16:**
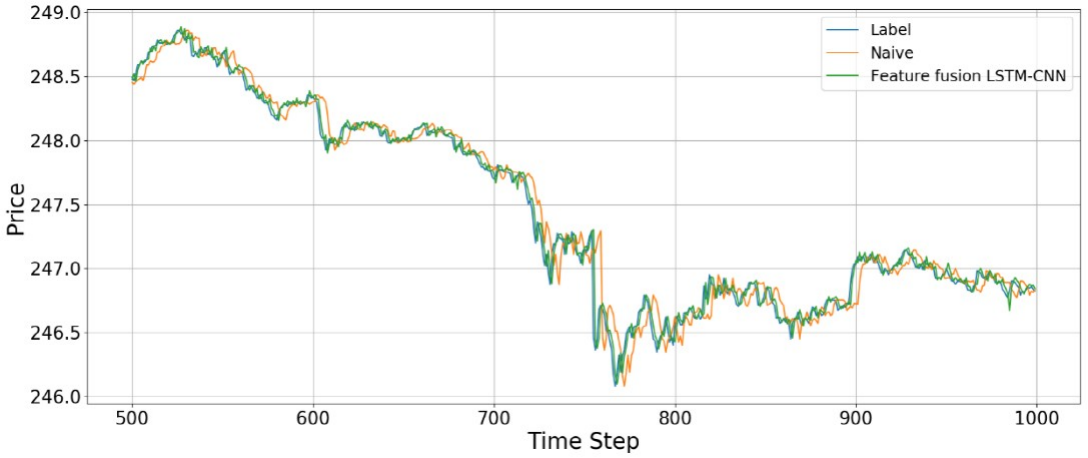
An example of predicting stock prices using the feature fusion LSTM-CNN model with a test dataset of between 500 and 1,000 data points. The input data are candlebar chart and stock time series.

**Fig 17:**
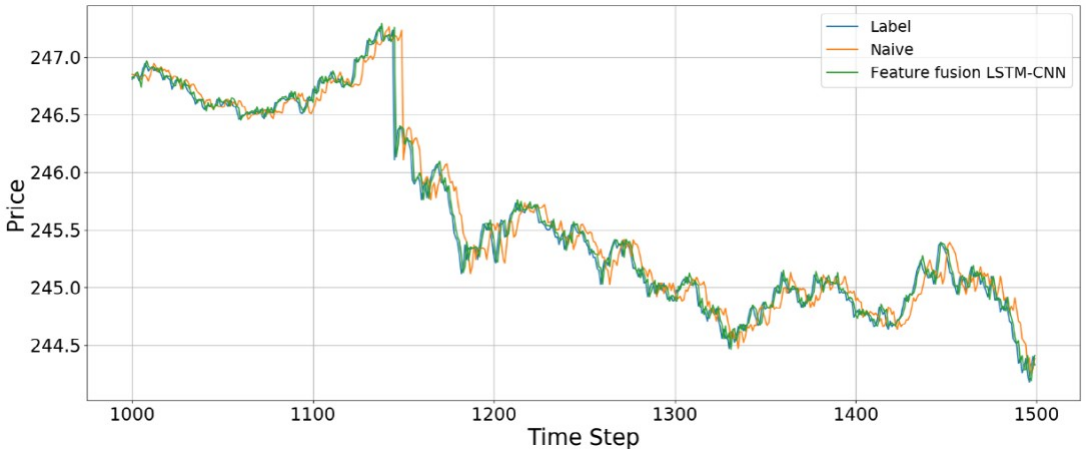
An example of predicting stock prices using the feature fusion LSTM-CNN model with a test dataset of between 1,000 and 1,500 data points. The input data are candlebar chart and stock time series.

**Fig 18:**
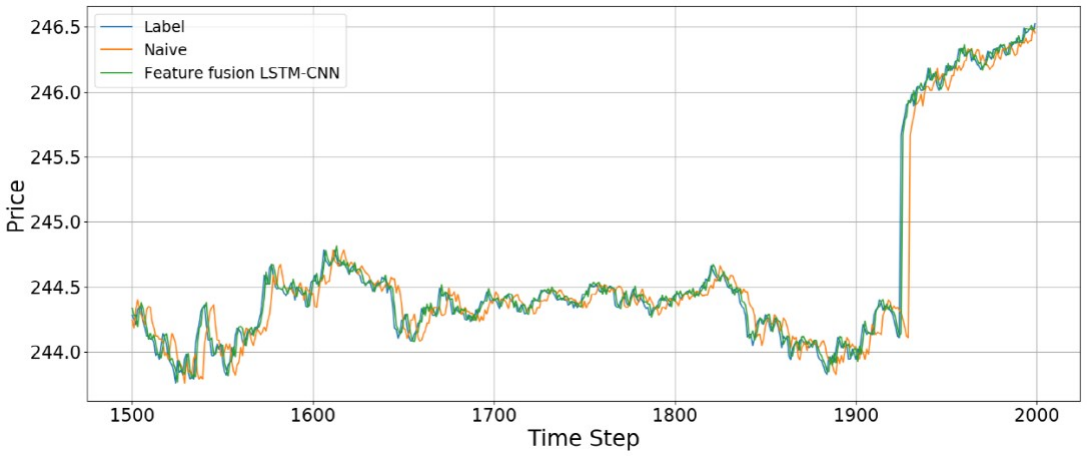
An example of predicting stock prices using the feature fusion LSTM-CNN model with a test dataset of between 1,500 and 2,000 data points. The input data are candlebar chart and stock time series.

Fig 14 shows the prediction results using the out-of-sample data for the feature fusion LSTM-CNN model using the candlebar chart, which is the best of the chart images, and stock time series data. In the out-of-sample dataset, the time interval is from August 1, 2017, to October 16, 2017, comprising 19,474 data points. As we see in Fig 14, it seems like the predicted prices fit actual prices. However, since we plotted all of the out-of-sample dataset in one figure, it can be seen that predicted price follows actual price well. Thus, we divide the whole period into 500 time steps per figure to see a more detailed movement in the predictions of the feature fusion LSTM-CNN model. Therefore, we estimate the out-of-sample predictions with 500 time steps at a time and compare our proposed feature fusion LSTM-CNN model using candlebar chart and stock time series with the naive model. We produce 39 figures since the testing dataset has 19,474 data points. Figs 15 to 18 show an example of predicting stock prices movements with the out-of-sample dataset between 0 to 2,000. The remaining figures are in Supporting Information.

In this study, we make different representations of stock price data to predict stock price. Theoretically, stock price follows a random process. According to the Markov process, *P*_*t*_ is greatly influenced by *P*_*t*−1_ where *P*_*t*_ and *P*_*t*−1_ are a stock price at time *t* and *t* − 1. Therefore, the graph shape of the data predicted at *P*_*t*+5_ is affected by the data at the previous time point, *P*_*t*_. In this study, it can be confirmed that the predicted term, *P*_*t*+5_, seems to be lagged particularly when plotted against the graph of *P*_*t*_. We would like to note that the stock price data themselves have a better predictive ability than each of the models considering the time-series characteristics of the stock price data. However, this study did not prevent the phenomenon of being lagged because only one stock price data was used. To remove this characteristic, LSTM input was used to remove the noise by adopting the log profit rate rather than the stock price data, but this alone is considered insufficient. If we add noise-canceling methods such as autoencoder or wavelet transformation, we can expect better performance. In addition, if we add macro variables affecting SPY data to the time-series data or add technical indicators to the image, we can reduce the lagged phenomenon and reduce the error with the target values.

## Conclusion

In this study, we propose a feature fusion LSTM-CNN model for forecasting stock prices by combining features of different representations of financial time series data. In order to create the proposed model, we proceed as follows. First, we construct an SC-CNN model that is optimized for stock chart images. We construct four stock chart images to see which chart images perform better. We find that the candlestick chart performs the best since it has more information compared with other stock chart images. Additionally, we confirm that bar chart images that are reconstructed using trading volume data are important in predicting stock prices. An F-line chart has lower predictive value compared to a line chart. Thus, including more information in input data can improve predictive power. Second, we perform an experiment by adding volume information to stock price information using the early fusion method. As a result, we confirm that performance measures, such as RMSE, RMAE, and MAPE, decrease for all three fusion chart images, but their respective ranks are still maintained. Third, we construct an ST-LSTM model that is optimized for a stock time series that includes the log returns of close prices and trading volumes. We confirm that the performance of the ST-LSTM model is better than that of the SC-CNN model. Lastly, we construct a feature fusion LSTM-CNN model combining the SC-CNN and ST-LSTM models with both fusion chart image and time series data. To improve the performance of the feature fusion LSTM-CNN model, we adjust the joint training by taking three losses that are constructed by the SC-CNN, ST-LSTM, and feature fusion LSTM-CNN models to reflect the information of each model. The performances of the feature fusion LSTM-CNN model are 0.098 (RMSE), 0.2291 (RMAE), and 0.0209 (MAPE), which are 22.09%, 20.89%, and 38.17% less, respectively, than those of the SC-CNN model and 18.18%, 17.56%, and 32.87% less, respectively, than those of the ST-LSTM model. Therefore, we confirm that the feature fusion LSTM-CNN model performs better than the single models (SC-CNN and ST-LSTM).

There is a way to further improve the performance of our proposed model. Adding technical indicators to the chart image can improve the performance of the model because more information can then be extracted from the image. Furthermore, if we add a new representation corresponding to a financial time series, such as a news article, we can be more efficient in predicting stock prices. The contribution of this study is that it is efficient to reduce the prediction error by using a combination of temporal and image features from the same data instead of using these features separately.

## Supporting information

S1 Fig Displays an example of stock chart images in validation dataset. (PDF)

S2 Fig Displays an example of fusion chart images in validation dataset. (PDF)

S3 Fig Displays an example of stock chart images in testing dataset. (PDF)

S4 Fig Displays an example of fusion chart images in testing dataset. (PDF)

S5 Fig Displays an example of predicting stock prices using the feature fusion LSTM-CNN model with a test dataset of between 2000 and 4000 data points. (PDF)

S6 Fig Displays an example of predicting stock prices using the feature fusion LSTM-CNN model with a test dataset of between 4000 and 6000 data points. (PDF)

S7 Fig Displays an example of predicting stock prices using the feature fusion LSTM-CNN model with a test dataset of between 6000 and 8000 data points. (PDF)

S8 Fig Displays an example of predicting stock prices using the feature fusion LSTM-CNN model with a test dataset of between 8000 and 10000 data points. (PDF)

S9 Fig Displays an example of predicting stock prices using the feature fusion LSTM-CNN model with a test dataset of between 10000 and 12000 data points. (PDF)

S10 Fig Displays an example of predicting stock prices using the feature fusion LSTM-CNN model with a test dataset of between 12000 and 14000 data points. (PDF)

S11 Fig Displays an example of predicting stock prices using the feature fusion LSTM-CNN model with a test dataset of between 14000 and 16000 data points. (PDF)

S12 Fig Displays an example of predicting stock prices using the feature fusion LSTM-CNN model with a test dataset of between 16000 and 18000 data points. (PDF)

S13 Fig Displays an example of predicting stock prices using the feature fusion LSTM-CNN model with a test dataset of between 18000 and 19474 data points. (PDF)
