# Supplementary material for: Forecasting stock prices with a feature fusion LSTM-CNN model using different representations of the same data
Source: PLoS One. 2019 Feb 15;14(2):e0212320. doi: 10.1371/journal.pone.0212320 (PMC6377125; doi:10.1371/journal.pone.0212320)
Supplement: S3 Fig — (PDF) [file pone.0212320.s003.pdf]

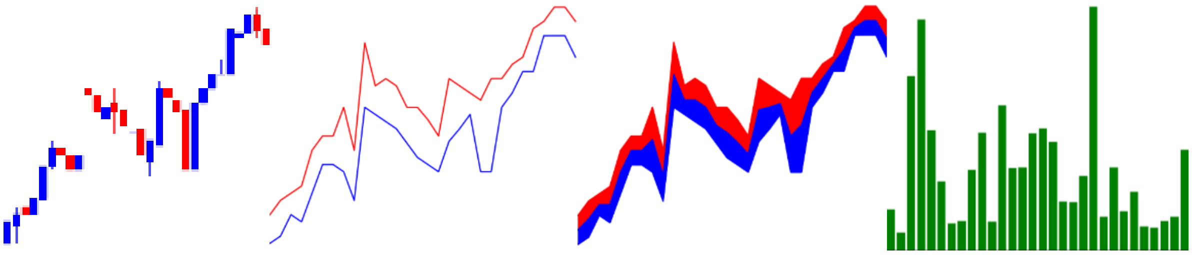

(a) (b) (c) (d)

Fig. An example of four stock chart images using SPY data in testing dataset.
(a) Candlestick chart, (b) Line chart, (c) F-line chart, and (d) Bar chart
