# Supplementary material for: Forecasting stock prices with a feature fusion LSTM-CNN model using different representations of the same data
Source: PLoS One. 2019 Feb 15;14(2):e0212320. doi: 10.1371/journal.pone.0212320 (PMC6377125; doi:10.1371/journal.pone.0212320)
Supplement: S4 Fig — (PDF) [file pone.0212320.s004.pdf]

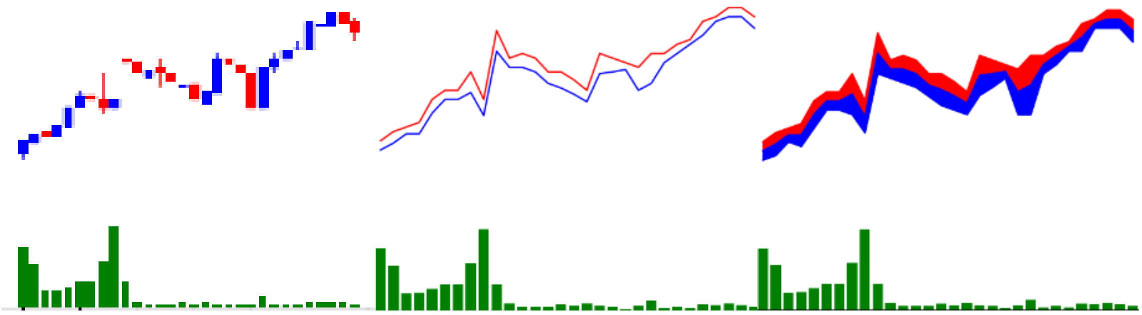

(a) (b) (c)

Fig. An example of four stock chart images using SPY data in testing dataset.
(a) Candlebar chart, (b) Linebar chart, and (c) F-linebar chart
