# Supplementary figures and images for: Forecasting stock prices with a feature fusion LSTM-CNN model using different representations of the same data
Source: PLoS One. 2019 Feb 15;14(2):e0212320. doi: 10.1371/journal.pone.0212320 (PMC6377125; doi:10.1371/journal.pone.0212320)

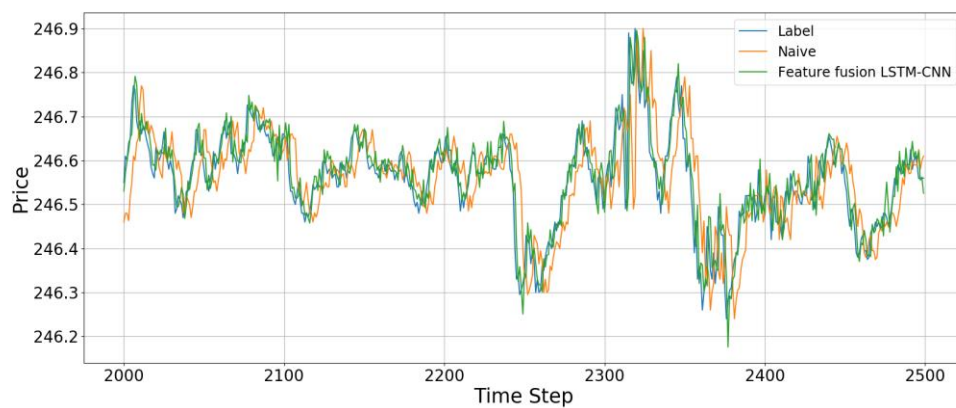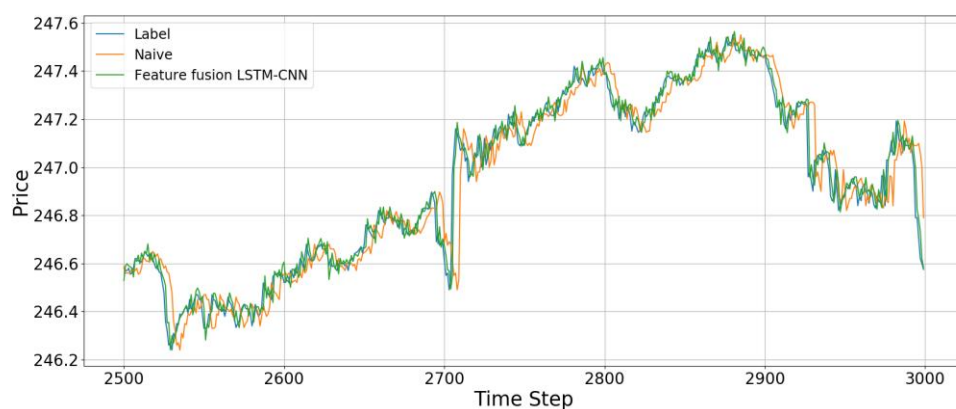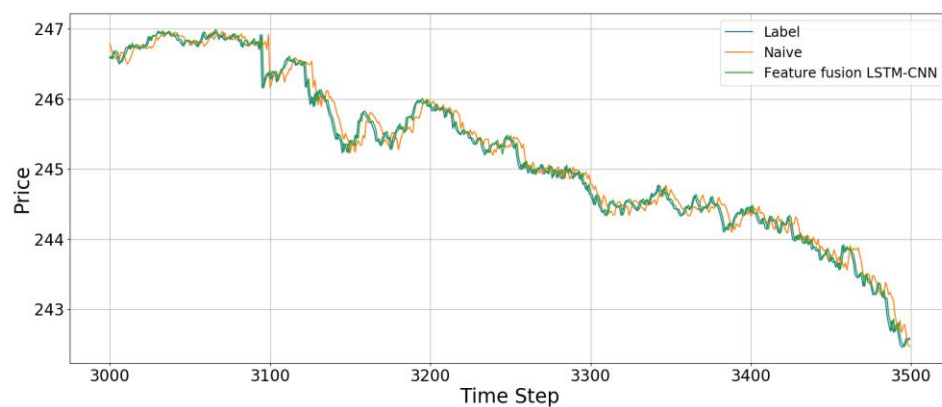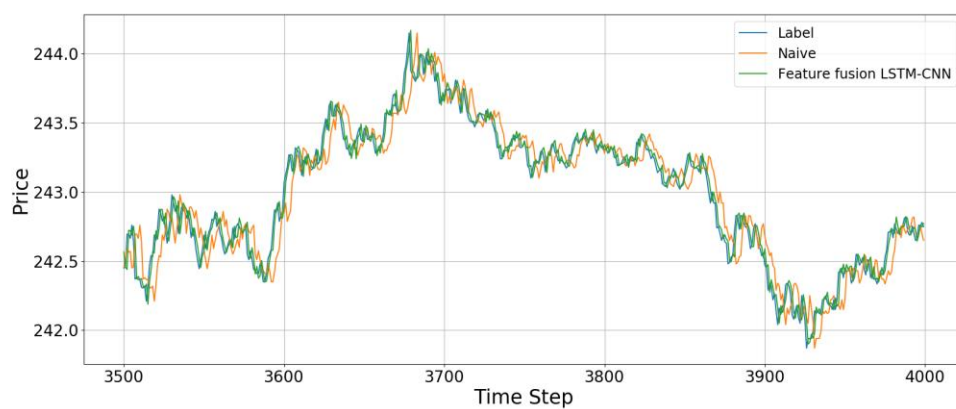

Supplement: S5 Fig — (PDF) [file pone.0212320.s005.pdf]

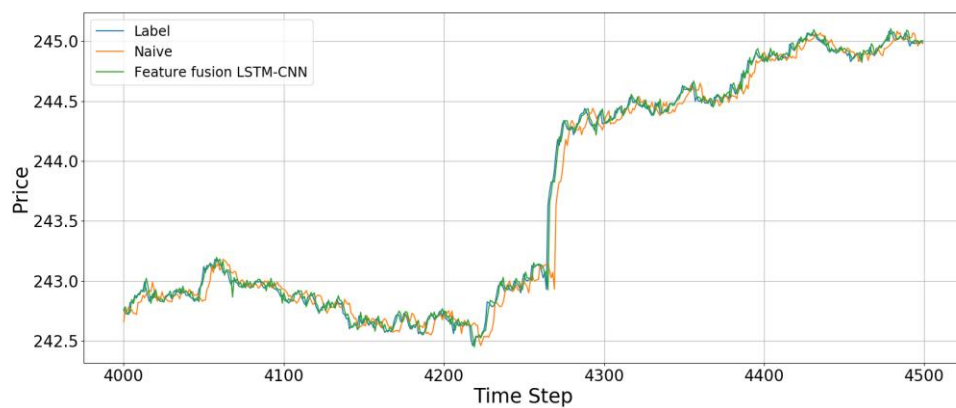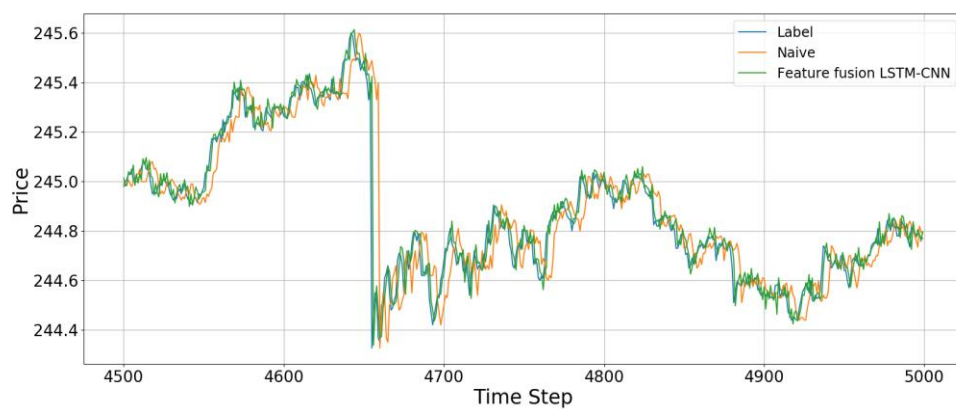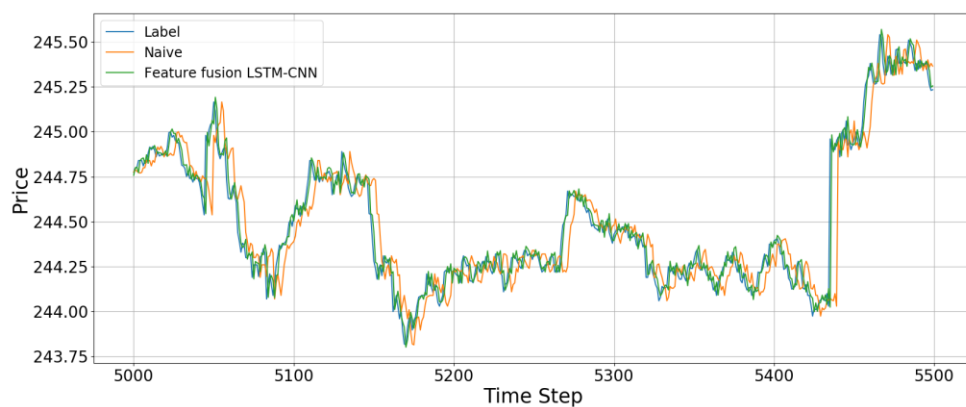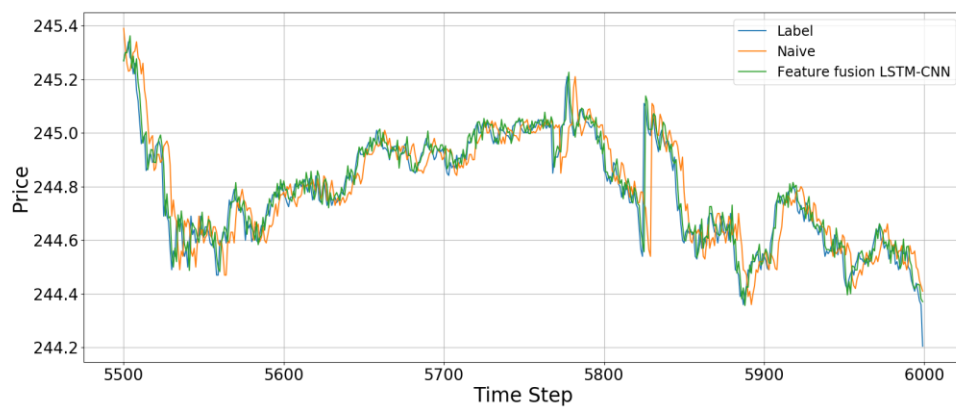

Supplement: S6 Fig — (PDF) [file pone.0212320.s006.pdf]

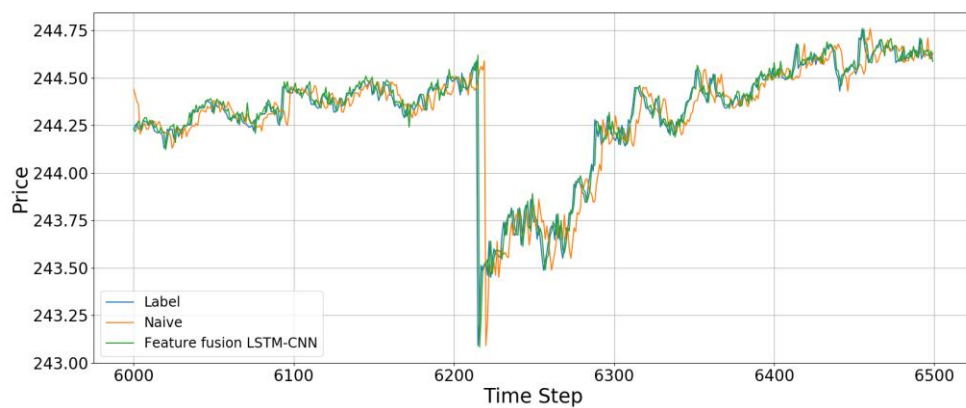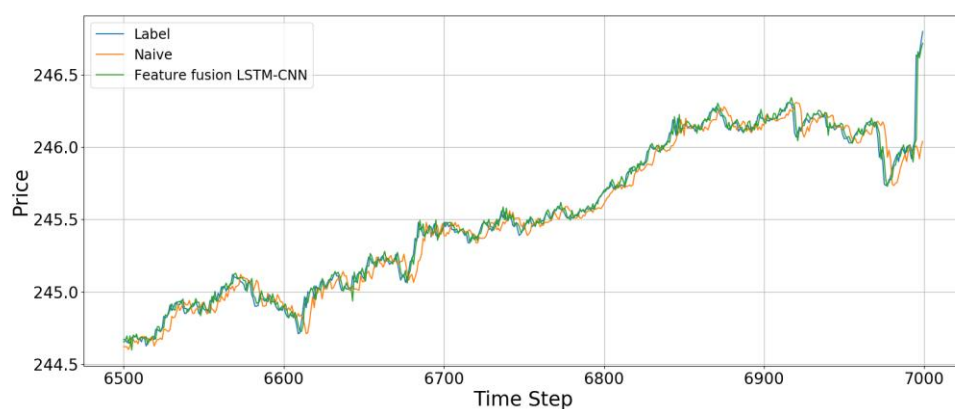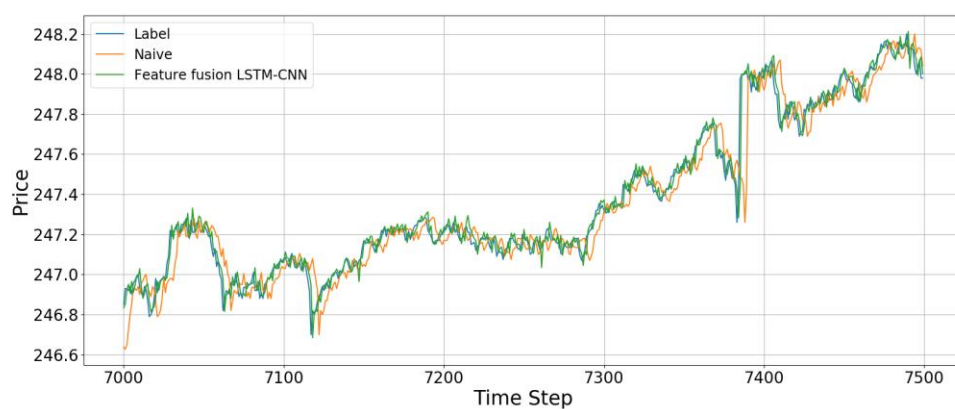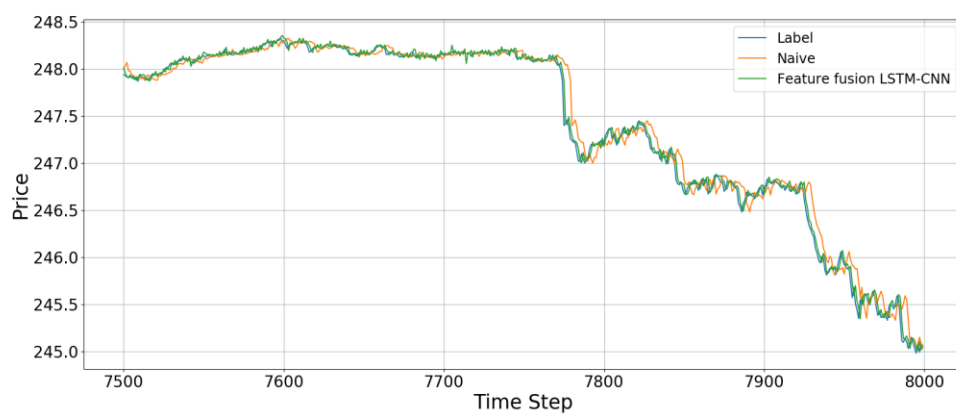

Supplement: S7 Fig — (PDF) [file pone.0212320.s007.pdf]

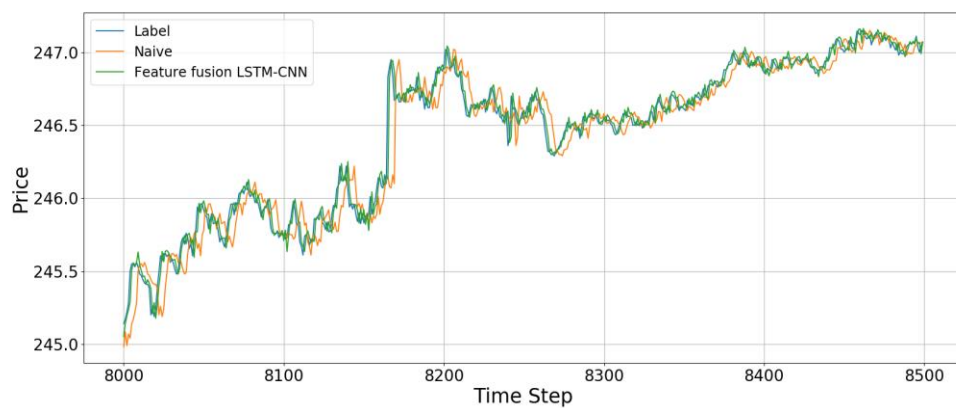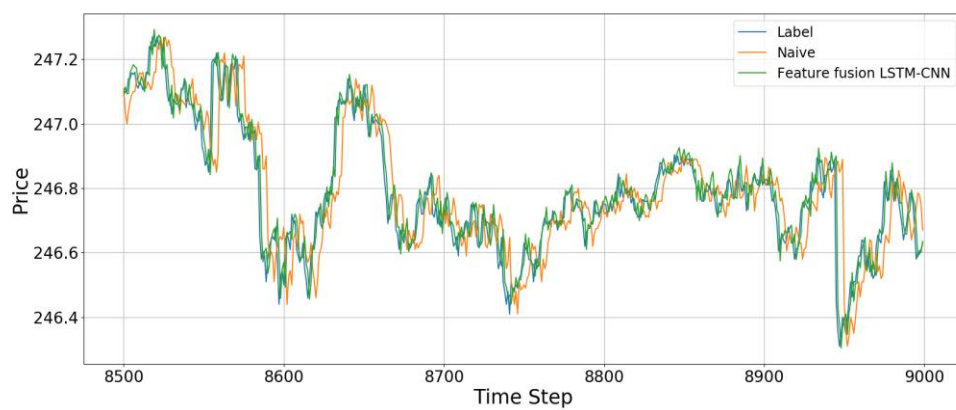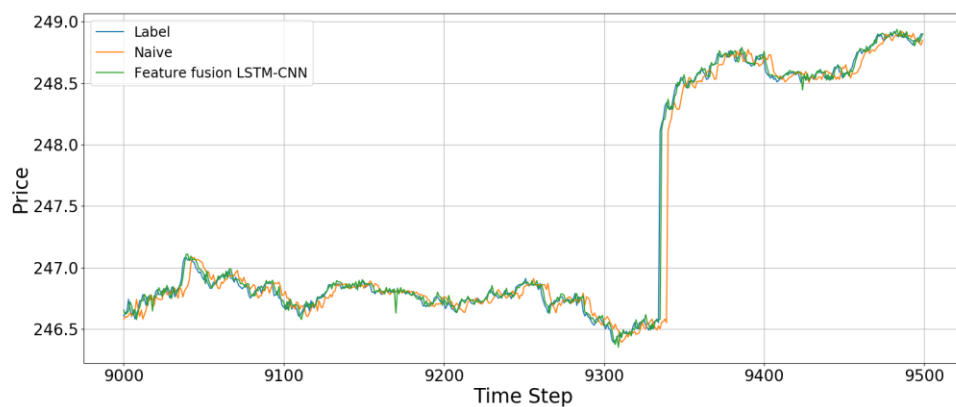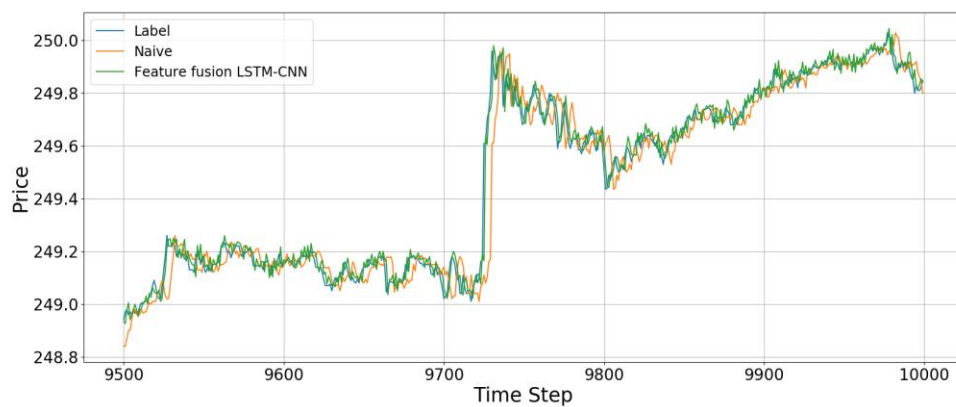

Supplement: S8 Fig — (PDF) [file pone.0212320.s008.pdf]

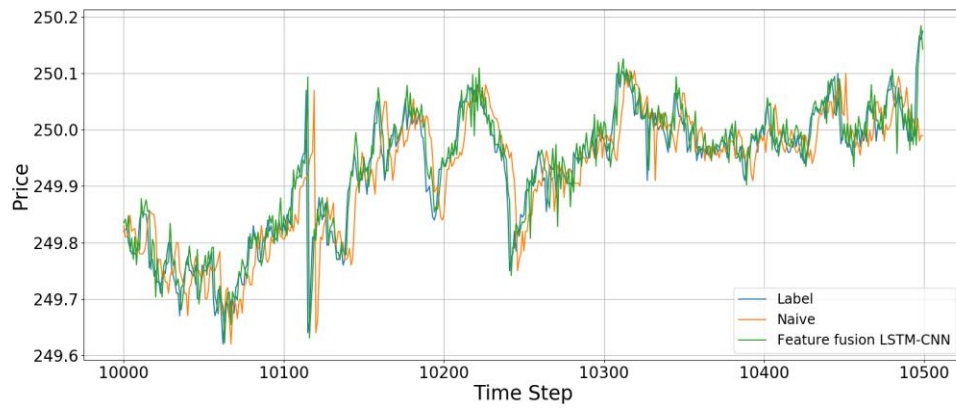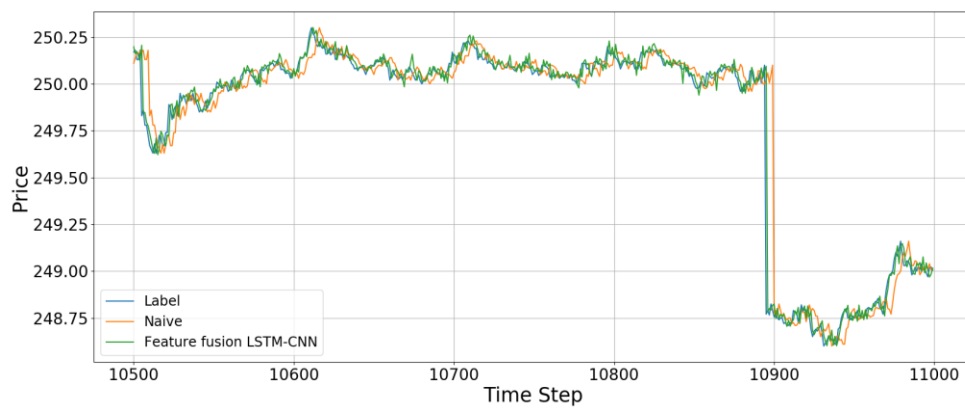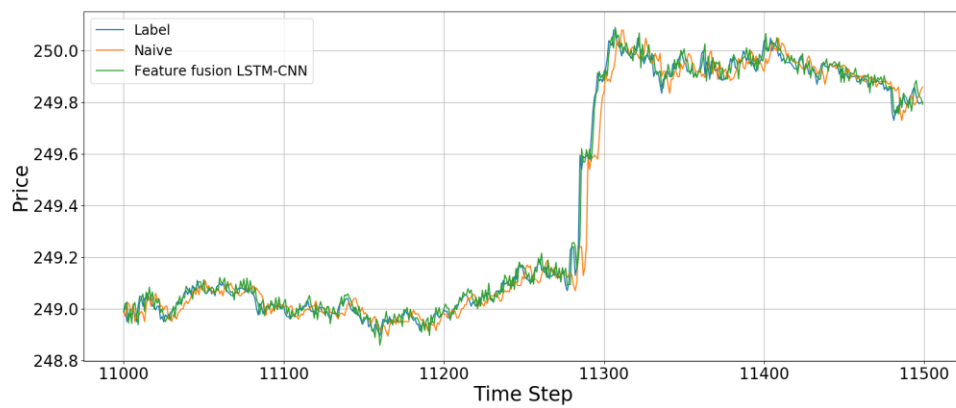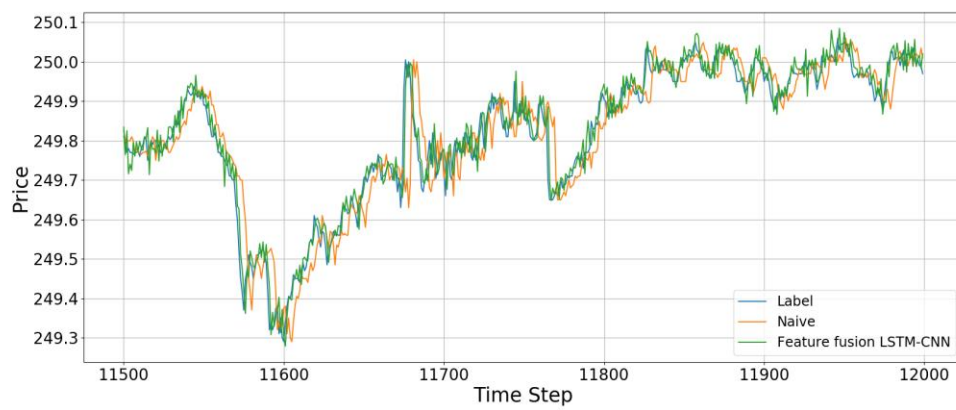

Supplement: S9 Fig — (PDF) [file pone.0212320.s009.pdf]

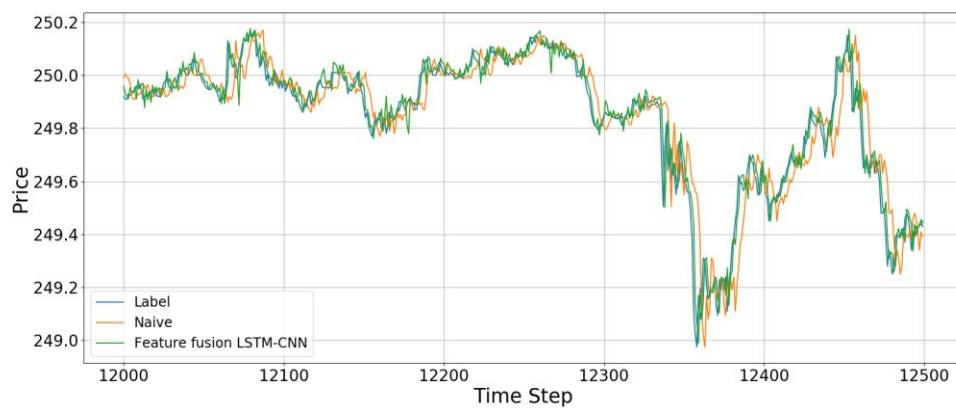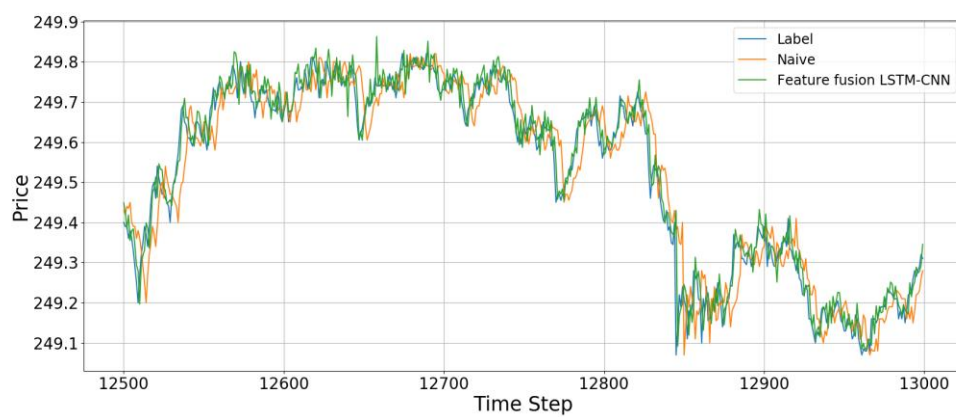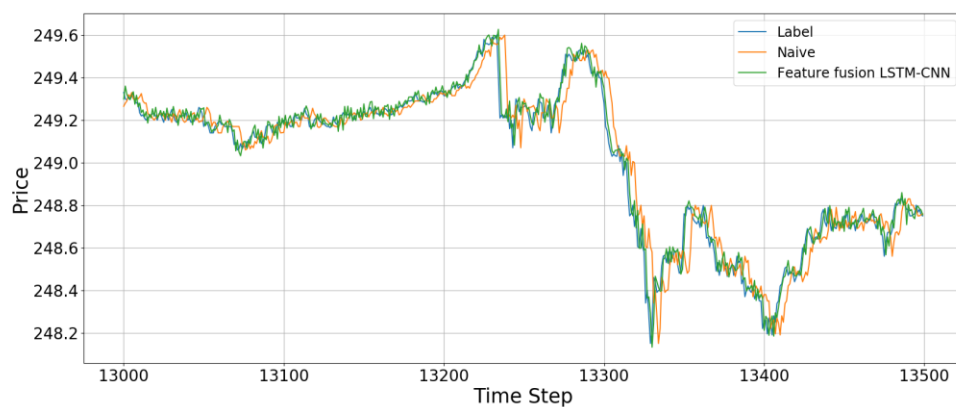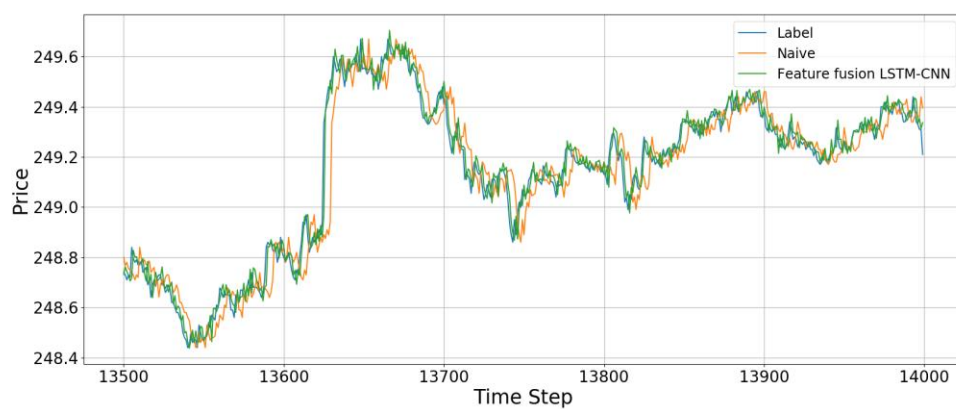

Supplement: S10 Fig — (PDF) [file pone.0212320.s010.pdf]

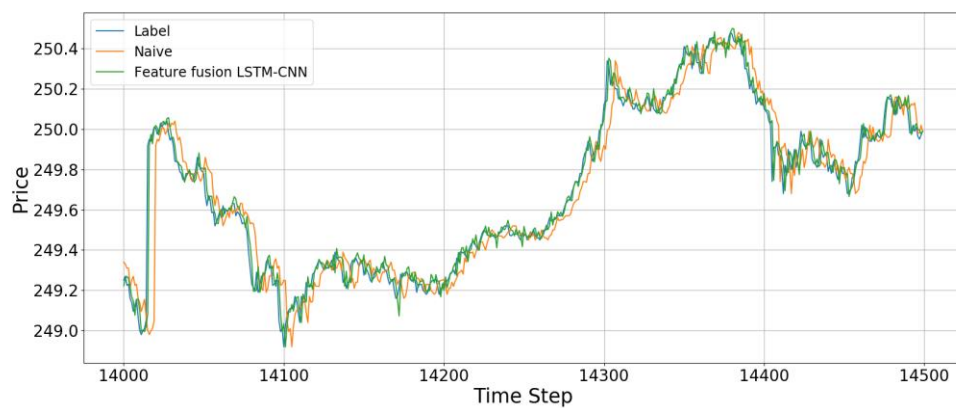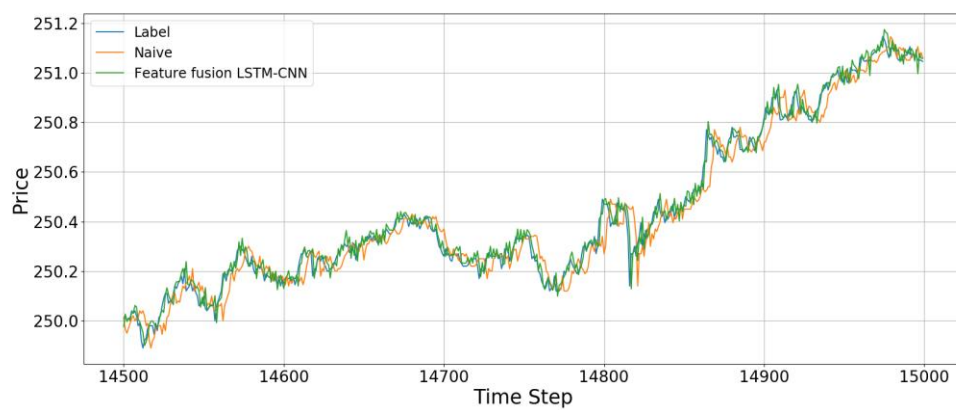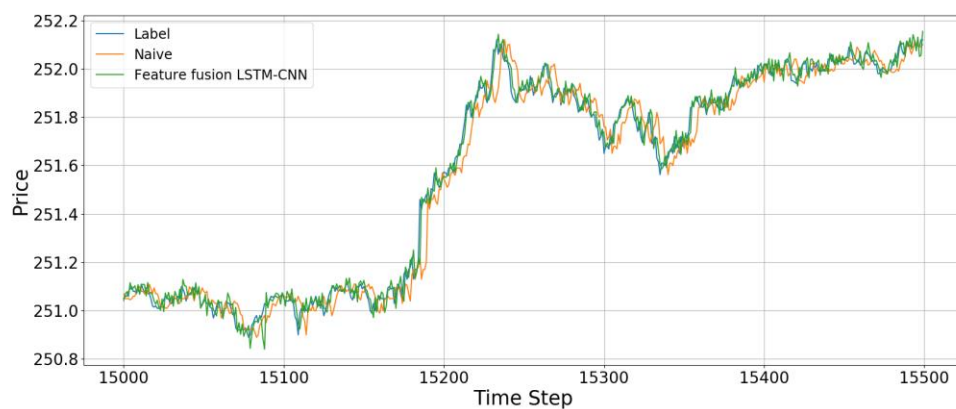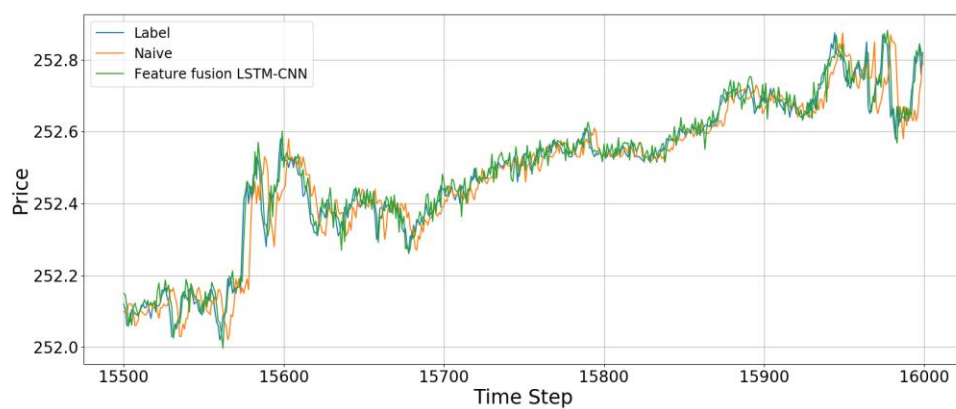

Supplement: S11 Fig — (PDF) [file pone.0212320.s011.pdf]

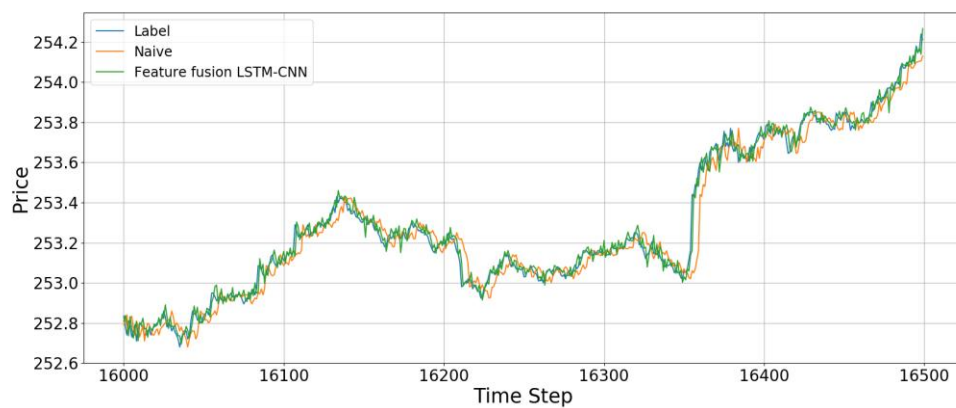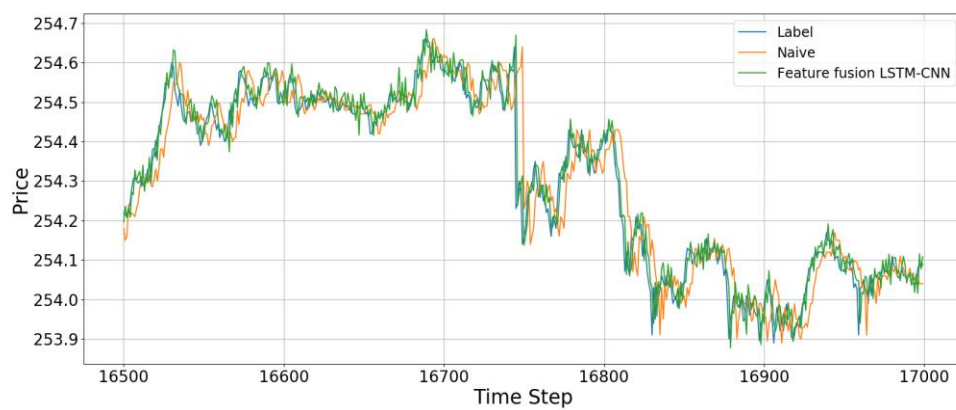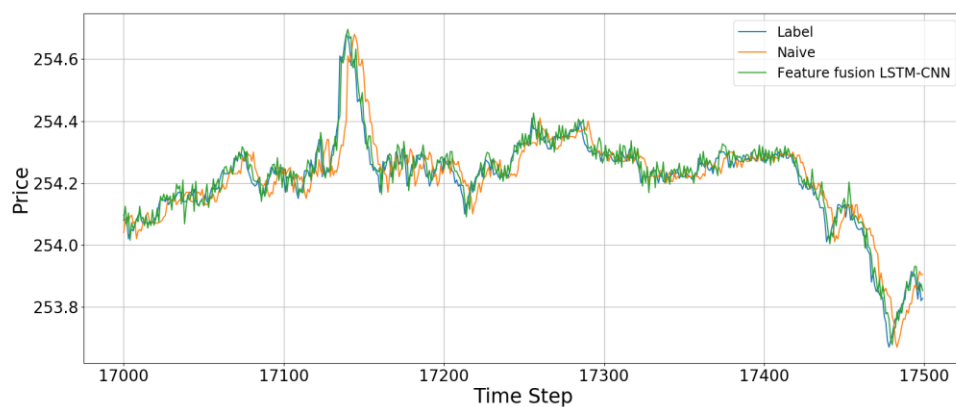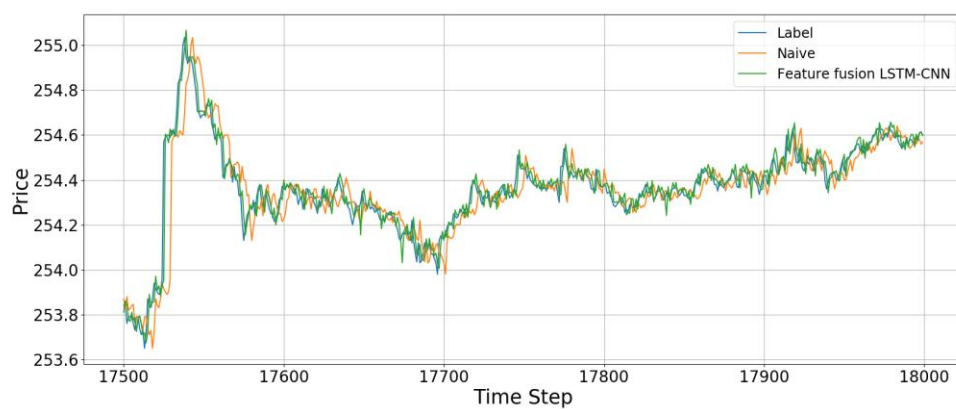

Supplement: S12 Fig — (PDF) [file pone.0212320.s012.pdf]

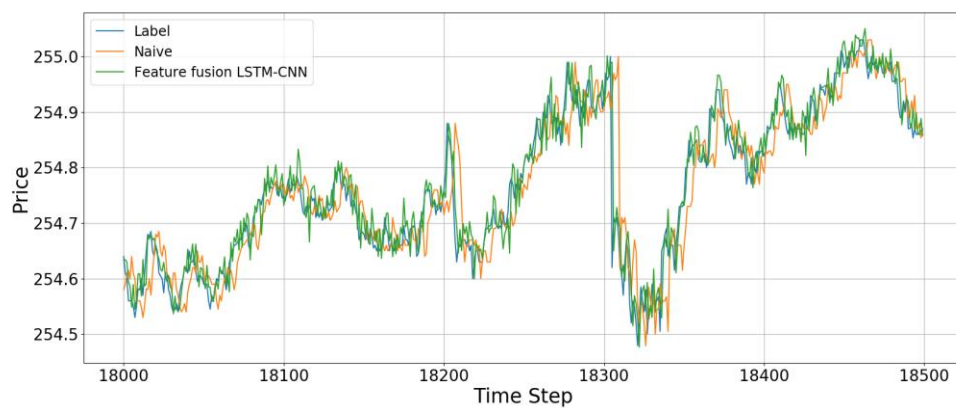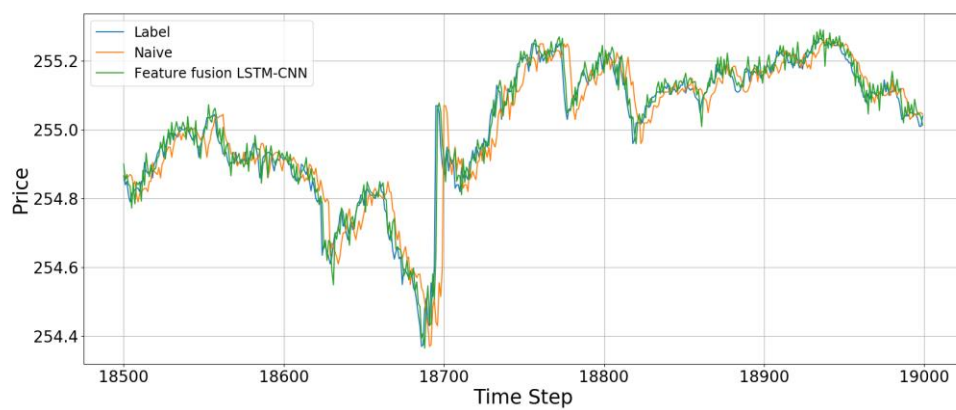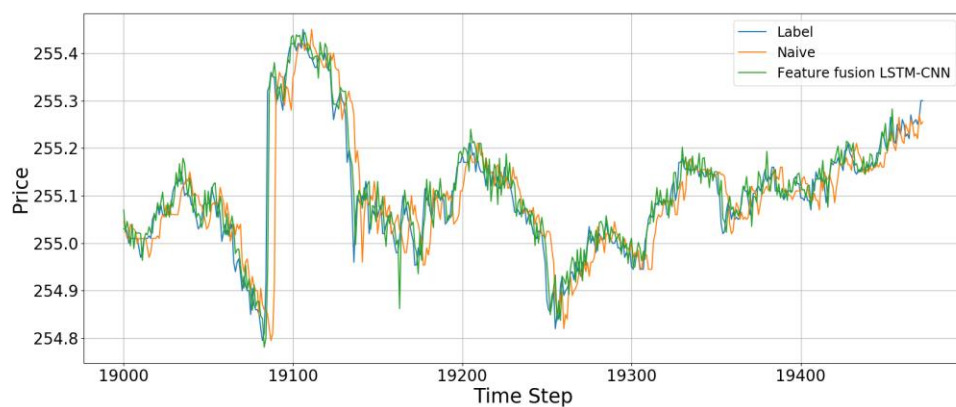

Supplement: S13 Fig — (PDF) [file pone.0212320.s013.pdf]
